# Persistent Photoluminescence Superlinearity Beyond Static Recombination Physics in Metal Halide Perovskites

**DOI:** 10.1002/advs.78378

**Published:** 2026-10-18

**Authors:** Mehri Ghasemi, Qi Li, Baohua Jia, Xiaoming Wen

**Affiliations:** ^1^ School of Science Centre For Atomaterials and Nanomanufacturing RMIT University Melbourne Victoria Australia

**Keywords:** absorption (chemistry), chemical physics, excitation, material properties, materials science, optics, photoluminescence, physical chemistry, physics, thin film

## Abstract

Persistent intermediate power‐law exponent (β) values in metal halide perovskites commonly occur across excitation regimes in which assignment to a single carrier‐population recombination limit becomes ambiguous. Their strong dependence on excitation conditions further indicates that excitation‐dependent photoluminescence cannot always be interpreted uniquely through a static Shockley‐Read‐Hall (SRH)‐bimolecular‐Auger recombination picture. Here, power‐dependent and time‐resolved photoluminescence measurements on methylammonium lead iodide (MAPbI_3_, MA = CH_3_​NH_3_) thin films reveal correlated evolution of excitation scaling, delayed emission, and illumination‐history response under conditions that systematically modify vibrational dissipation and carrier‐relaxation pathways. These behaviors are described using a dual‐channel framework in which transient vibrational energy retention contributes to delayed radiative response. The results support an interpretation of PL superlinearity as an emergent, condition‐dependent response associated with coupled carrier‐lattice relaxation dynamics, rather than a simple transition between fixed recombination‐order limits. More broadly, these results show that β and photoluminescence quantum yield (PLQY) should not be treated as fixed intrinsic material constants, but as excitation‐conditioned observables shaped by the evolving relaxation environment, highlighting limitations in interpreting PL scaling laws as direct indicators of recombination pathways in soft semiconductors.

## Graphical abstract

Persistent intermediate photoluminescence superlinearity in metal halide perovskites challenges the conventional interpretation of β as a fixed recombination‐order indicator. Correlated steady‐state and time‐resolved measurements support a condition‐dependent carrier–lattice relaxation framework, suggesting that β and photoluminescence quantum yield evolve with the excitation‐dependent relaxation environment.

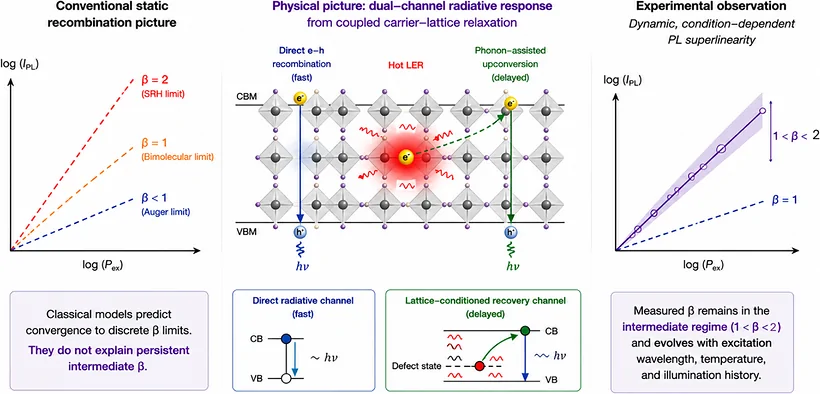

## 1 Introduction

Metal halide perovskites (MHPs) have emerged as leading optoelectronic semiconductors because they combine strong optical absorption, long carrier diffusion lengths, tunable direct bandgaps, and comparatively low nonradiative recombination losses [1, 2]. Their high photoluminescence quantum yields (PLQYs), even in solution‐processed polycrystalline films, have made photoluminescence (PL) a central probe of recombination behavior, defect tolerance, and optoelectronic quality in perovskite absorbers and emitters [3, 4, 5, 6, 7]. A recurring feature of these materials is the superlinear dependence of PL intensity on excitation fluence, commonly expressed as 𝐼_PL_∝𝑃^𝛽^, where the power‐law exponent β exceeds unity (β  >  1) [8, 9, 10, 11, 12, 13]. The exponent β carries direct physical significance because it is commonly associated with recombination order and is used to infer the dominant carrier‐relaxation regime. Its interpretation affects the analysis of trap‐assisted recombination, radiative efficiency, quasi‐Fermi‐level splitting, and nonradiative losses [6, 7]. However, reported β values in MHPs vary widely across compositions, morphologies, fabrication routes, and measurement conditions [8, 9, 10, 11, 12, 13]. Even nominally similar MAPbI_3_ systems exhibit markedly different intermediate exponents, while simplified analyses often adopt an approximately fixed value, frequently close to β ≈ 1.5, to represent superlinear behavior within a selected excitation regime [14]. This discrepancy indicates that the variability of β reflects unresolved aspects of the excitation‐dependent radiative response.

Within classical semiconductor recombination theory, superlinear PL can arise under specific limiting conditions. In the low‐injection regime, first‐order Shockley‐Read‐Hall (SRH) or surface‐related recombination can control the free‐carrier population while radiative emission remains bimolecular, giving β ≈ 2 [15, 16]. As excitation increases and bimolecular recombination becomes dominant, the response approaches β ≈ 1, while Auger recombination drives sublinear behavior at sufficiently high carrier density. Additional descriptions involving trap filling, defect‐assisted recombination, photon recycling, free‐carrier redistribution, and carrier‐lattice coupling have been developed to account for nonlinear PL in MHPs [8, 10, 17, 18, 19, 20, 21]. These mechanisms describe important regimes of perovskite photophysics. Between these asymptotic limits, competition among first‐ and second‐order processes can generate intermediate effective slopes, commonly within 1.1< β < 1.8, and trap filling or distributed trapping can further broaden the crossover regime. What remains unresolved is why intermediate β values in MHPs remain strongly condition‐dependent and frequently evolve together with other signatures of the photoexcited relaxation dynamics, rather than behaving solely as a carrier‐density‐driven crossover between established recombination channels [8, 9, 10, 11, 12]. The persistence and variability of intermediate β values suggest that the measured PL response cannot be fully interpreted solely as a transition between fixed recombination orders. MHPs are soft, anharmonic semiconductors with strong carrier‐lattice coupling, dynamic disorder, low thermal conductivity, and pronounced phonon‐mediated relaxation [22, 23, 24, 25, 26, 27, 28, 29, 30, 31, 32, 33]. These properties influence carrier localization, trapping, detrapping, thermalization, and hot‐carrier cooling. Recent work has also shown that shallow defects and nonequilibrium relaxation processes can generate continuously evolving PL dynamics that are not uniquely resolved by conventional lifetime or recombination‐order descriptions [24]. The excitation dependence of PL scaling is consistent with radiative response evolving together with the nonequilibrium relaxation environment established during excitation. This work addresses that question by examining whether persistent PL superlinearity can be interpreted as a nonequilibrium radiative response shaped by coupled carrier‐lattice relaxation dynamics. The analysis suggests that the condition‐dependent evolution of β and radiative efficiency evolves together with the transient energetic state established within the excited soft lattice, challenging interpretation of excitation‐dependent PL solely through fixed recombination‐order transitions or static carrier‐population kinetics.

## 2 Conceptual Challenges and Physical Framework

### 2.1 Limitations of Existing Interpretations of PL Superlinearity

The broad distribution of reported β values in MHPs (Table 1) reflects a central ambiguity in the interpretation of excitation‐dependent PL. Existing mechanisms, including trap saturation [8, 10], free‐carrier dynamics [34, 35], Auger processes [22], photon recycling [4, 36], and carrier‐lattice coupling effects [21, 37], account for important components of nonlinear PL behavior. What remains unresolved is the persistent appearance of intermediate β values across excitation regimes in which convergence toward the classical limiting slopes is not observed, together with their strong dependence on spectral, thermal, and illumination conditions.

**TABLE 1:** Reported superlinear photoluminescence power‐law exponents (β) in metal halide perovskites across different compositions, morphologies, and fabrication approaches, highlighting the widespread occurrence and variability of excitation‐dependent nonlinear radiative behavior in these materials.

Composition	Superlinear Coefficient(β)	Remarks
MAPbI_3_	1.515 and 1.173	Thick nanoplatelet, ∼210/215 nm [13]
MAPbI_3_	1.547 and 1.119	Thin nanoplatelet, ∼21 nm [13]
MAPbI_3_	1.47	Single crystal [8]
FAPbI_3_	1.49	Thin film [8]
MAPbI_3_	1.575	Thin film [9]
FAPbI_3_	1.815	Thin film [9]
FA_0.2_MA_0.8_PbI_3_	1.570	Thin film [9]
CsPbBr_3_	1.60	Thin film [40]
5% Li‐CsPbBr_3_	1.1	Li‐doped spin‐coated thin film [40]
MAPbI_3_	1.619	Thin film, Spin coating fabrication [10]
MAPbI_3_	1.82	Gas‐assisted fabrication [10]
MAPbI_3_	1.39‐1.67	Single crystals, before and after treatment with CsI top‐layer [12]
MaPbI_3_	1.3‐1.63	Thin film on glass; β varies systematically with excitation conditions (This work)

Trap‐assisted and defect‐mediated recombination models provide a natural explanation for superlinear PL in regimes where trap populations evolve with carrier density [8, 10]. At low excitation density, trap‐mediated losses suppress radiative emission; as traps become progressively occupied, the radiative contribution increases, and the apparent PL scaling becomes superlinear. This mechanism captures crossover behavior between defect‐limited and bimolecular regimes and explains the sensitivity of PL scaling to defect density. Such models can generate intermediate apparent slopes as trap occupation and the balance between first‐ and second‐order recombination evolve with carrier density. The remaining ambiguity is therefore not the existence of intermediate β itself, but whether its condition dependence can be described independently of the concurrent evolution of the relaxation dynamics. Assignment to trap occupation or defect‐mediated recombination identifies the carrier‐population process involved, but does not by itself specify how the relaxation environment governing that process evolves with excitation conditions or illumination history. In the present measurements, changes in β are accompanied by systematic changes in the delayed emissive component and by illumination‐history dependence, indicating that the effective recombination landscape does not remain invariant across the measurement conditions [14, 22]. In combined SRH‐bimolecular‐Auger descriptions, apparent intermediate slopes can arise over restricted excitation ranges as different recombination terms compete. However, Auger recombination is intrinsically nonradiative and becomes important primarily at high carrier density, where it suppresses PL intensity and drives the response toward sublinear scaling. Persistent radiative superlinearity under moderate excitation conditions in MHP thin films and single crystals is therefore not naturally described as an Auger‐dominated effect. Photon recycling and optical reabsorption can increase apparent PL intensity and lengthen emission dynamics in high‐quality perovskite absorbers [4, 36]. These processes are important in efficient films because emitted photons can be reabsorbed and re‐emitted before escaping the material. Nevertheless, photon recycling modifies photon transport after radiative recombination has occurred. It does not define the microscopic origin of the excitation‐dependent scaling exponent, nor does it by itself explain why β varies across conditions that alter the carrier relaxation environment. Surface and interface recombination can also modify apparent recombination order [38], particularly in devices and heterostructures where contacts, transport layers, or interfacial defect populations introduce additional first‐order loss channels. Such effects are essential for device‐level analysis, but they cannot provide a general explanation for intermediate β values observed in contact‐free films and single crystals. The persistence of superlinearity in optically excited neat absorbers indicates that the phenomenon is not solely a consequence of interfacial recombination. The common feature of these interpretations is that they describe individual carrier‐relaxation processes operating within a prescribed recombination landscape. Trap filling, surface recombination, photon recycling, carrier redistribution, and related mechanisms each capture important aspects of excitation‐dependent PL, yet they are generally invoked independently to explain specific experimental observations. This approach provides valuable descriptions of individual processes but does not explicitly consider whether the relaxation environment governing these processes may itself evolve under different excitation conditions.

A central limitation of existing interpretations is that excitation‐dependent PL superlinearity is still analyzed predominantly through fixed carrier‐population recombination schemes, despite the strongly nonequilibrium nature of photoexcited MHP lattices. Under optical excitation, carrier relaxation occurs in the presence of strong electron‐phonon coupling, dynamic disorder, anharmonic lattice motion, and slow vibrational dissipation [21, 34, 35, 37, 39]. Recent studies have further shown that shallow defects and nonequilibrium relaxation processes can generate continuously evolving PL dynamics that are not always uniquely resolved within conventional recombination analyses [24]. The energetic environment governing recombination can therefore evolve during excitation rather than remaining a static background to carrier kinetics. Persistent intermediate β values, particularly in regimes where neither an ideal SRH‐to‐bimolecular crossover nor strong Auger quenching provides a complete description, point to a missing connection between carrier recombination and the transient storage, redistribution, and dissipation of excitation energy in the soft perovskite lattice.

### 2.2 Nonequilibrium Lattice Contribution to Radiative Response

Optical excitation in MHPs generates free carriers while also transferring energy to the lattice through carrier‐phonon interactions. In conventional recombination analysis, this lattice energy is usually treated as a dissipative background, while PL is described through the evolution of carrier populations among SRH, bimolecular, Auger, surface‐mediated, or photon‐recycling pathways. This approximation becomes incomplete when the lattice itself evolves on timescales that overlap with carrier relaxation and recombination. In soft perovskites, anharmonic lattice motion, low thermal conductivity, strong electron‐phonon coupling, and local structural fluctuations can slow the dissipation of vibrational energy and create nonequilibrium lattice environments under illumination [41, 42, 43, 44, 45, 46, 47, 48]. Such nonequilibrium environments can modify the energetic landscape encountered by relaxed or weakly localized carriers. Carriers occupying shallow subgap states are not necessarily removed irreversibly from the emissive population; their probability of returning to radiative pathways depends on the local balance between trapping, detrapping, energy dissipation, and carrier‐phonon exchange. If part of the vibrational energy generated during relaxation persists locally, the PL response becomes sensitive to the transient lattice state as well as to the instantaneous free‐carrier density.

We refer to these transient nonequilibrium lattice environments as lattice energy reservoirs (LERs), used here as a phenomenological description of spatially heterogeneous nonequilibrium lattice states generated through carrier‐phonon energy transfer and subsequent anharmonic lattice relaxation [42, 43, 49]. Within this framework, the LER describes an evolving lattice environment involving coupled vibrational and structural degrees of freedom, rather than a fixed population of long‐lived phonons or an electronic trap state. Energy deposited into the lattice can be redistributed among vibrational modes and structural degrees of freedom, including elastic potential energy associated with local lattice distortion or deformation, while remaining coupled to the electronic system as the photoexcited state evolves. Individual phonon relaxation and conventional thermal transport may therefore occur on substantially shorter timescales than the evolution of the collective excitation‐conditioned material state [27, 32, 33]. Weakly localized and shallowly trapped carriers constitute distinct electronic states whose trapping, recovery, and radiative recombination may be conditioned by this evolving relaxation environment. The resulting nonequilibrium lattice energy and structural response may consequently modify the relaxation conditions encountered by these carriers and influence their delayed recovery into emissive pathways (Figure 1) [42, 43, 49]. The LER framework therefore associates the lattice contribution to radiative relaxation with the evolving nonequilibrium energetic and structural state of the excited lattice, rather than with passive thermal‐energy confinement within a static domain. A more detailed discussion of the proposed LER formation process, associated phonon dynamics, and related experimental observations is provided in the Appendix S1.

**FIGURE 1:**
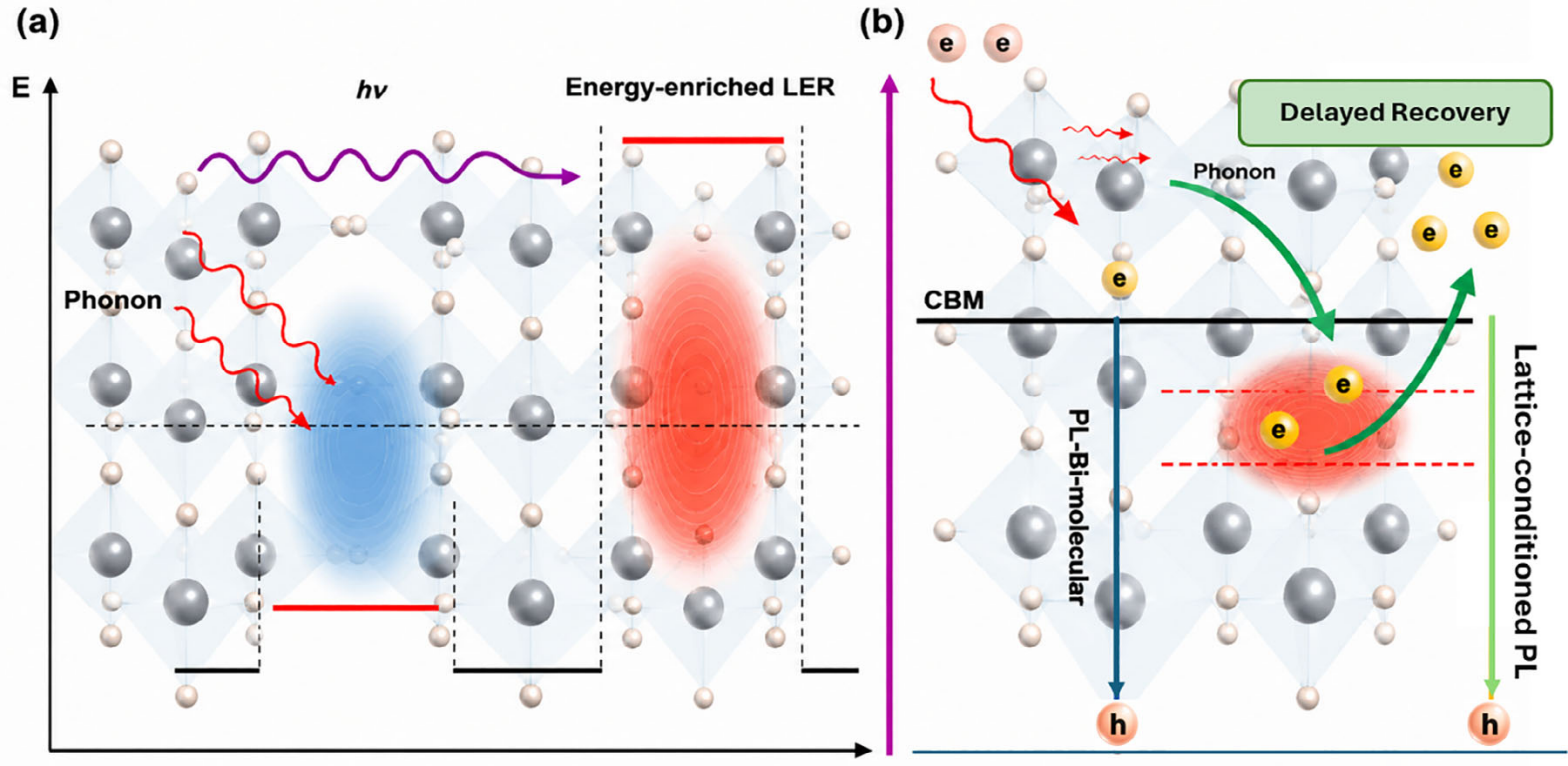
Conceptual framework of lattice‐conditioned radiative response in metal halide perovskites. (a) Schematic formation of a nonequilibrium lattice energy reservoir within the soft perovskite lattice. The LER is represented as a spatially heterogeneous nonequilibrium lattice environment associated with a dynamically distorted lattice region, where carrier‐phonon energy transfer, anharmonic redistribution, and local lattice relaxation modify the energetic environment experienced by relaxing carriers. The blue and red regions schematically represent progressively energy‐enriched nonequilibrium lattice configurations involving vibrational and structural degrees of freedom and are physically distinct from localized or trapped electronic states. (b) Schematic illustration of the proposed lattice‐conditioned radiative pathway. Within the proposed framework, the evolving nonequilibrium lattice environment can modify the relaxation conditions encountered by weakly localized or shallowly trapped carriers and thereby influence their delayed recovery into emissive states. The recovered carriers subsequently contribute to radiative electron‐hole recombination and, within the phenomenological description, contribute to the lattice‐conditioned component of the radiative response alongside the conventional bimolecular contribution. The relative weighting of the two contributions evolves with the nonequilibrium lattice state.

Related anomalously slow and illumination‐dependent phenomena reported in MHPs, including slowed hot‐carrier cooling, photobrightening, upconversion‐like emission, structural polarization, and dynamic defect tolerance, illustrate the broad sensitivity of carrier relaxation and radiative response to the evolving photoexcited state of these materials [25, 47, 50, 51, 52, 53, 54, 55, 56]. These phenomena can arise from different microscopic processes and are not necessarily governed by a single mechanism, but collectively demonstrate that the photoexcited state of MHPs cannot always be regarded as a static background to carrier recombination.

### 2.3 Dual‐Channel Radiative Description

A compact representation of this balance can be obtained by separating the measured PL into a conventional radiative component and an additional lattice‐assisted component within the experimentally relevant excitation range. The conventional steady‐state recombination balance is written as: *G_P_* =  *An* + *Bn* ^2^ + *Cn* ^3^ where *G_P_* is the photogeneration rate proportional to excitation intensity *P*, 𝑛 the steady‐state carrier density, and 𝐴, *B*, and 𝐶 represent effective first‐order, bimolecular, and Auger recombination coefficients, respectively. Within this framework, the conventional progression from β ≈ 2 toward β ≈ 1 and eventually sublinear behavior reflects transitions between established carrier‐population recombination regimes. The dual‐channel radiative framework used here extends this picture by introducing an additional radiative contribution associated with the nonequilibrium lattice environment. The total PL intensity is therefore written as:

$$I_{\mathrm{PL}}\left(P\right)=I_{\mathrm{PL},\mathrm{bi}}\left(P\right)+I_{\mathrm{PL}.\mathrm{Lattice}}\left(P\right)$$

where I_PL,bi_​(P)denotes the conventional radiative contribution and I_PL.Lattice_​(P) denotes the lattice‐assisted radiative contribution. In the excitation window analyzed here, this is expressed phenomenologically in the dual‐channel form:

$$I_{\mathrm{PL}}\;\left(P\right)=K_{1}P^{2}+\varepsilon K_{2}P^{\mu }$$

Here, *K*_1_and *K*_2_denote effective amplitudes of the two scaling terms, εdefines the excitation dependence of the additional contribution, and ε represents phenomenologically the state dependence of the lattice‐conditioned radiative contribution. The additional term describes how an evolving nonequilibrium lattice environment may contribute to the measured PL response. The parameters are not assigned as independently measured microscopic quantities, and their individual amplitudes are not used for quantitative comparison across experimental conditions. The relative contribution of the two terms varies with excitation power through their different excitation dependences.

In this phenomenological description, µ is expected to remain between the first‐ and second‐order limits when the additional contribution reflects delayed radiative recovery conditioned by the evolving lattice environment rather than direct bimolecular recombination of the entire free‐carrier population. The resulting PL scaling is governed by the coupled evolution of carrier recombination, trapping dynamics, and nonequilibrium lattice relaxation. Within this framework, changes in the nonequilibrium lattice state modify the relative weighting of the two radiative contributions, allowing the apparent PL scaling behavior to evolve under different excitation conditions. Changes in the relative weighting of the two radiative contributions therefore produce excitation‐dependent β values that do not converge to a single fixed recombination order. The apparent PL response consequently reflects the evolving nonequilibrium conditions established during excitation rather than a static recombination landscape governed solely by fixed carrier‐population recombination pathways.

## 3 Condition‐Dependent PL Superlinearity

### 3.1 Excitation Wavelength and Temperature Dependence

Power‐dependent PL measurements on MAPbI3 thin films reveal a persistent intermediate superlinear response whose exponent depends systematically on the excitation conditions. Representative PL spectra and intensity‐scaling behavior are shown in Figure 2, with the full spectral series and log‐log fitting data provided in Figures S1–S3. Across the measured excitation range, the extracted β values remain within the intermediate regime (Figure 2c,d), but they are not invariant for a given film. Instead, β changes reproducibly with both excitation wavelength and temperature, indicating that the apparent radiative scaling is coupled to the local recombination environment sampled during excitation. Each PL spectrum was acquired using a CCD integration time of 0.5 s, which was maintained throughout each power‐dependent measurement series. Spectra were collected sequentially from low to high excitation density, with an interval of approximately 2 s between successive acquisitions, during which the excitation was blocked to minimize cumulative illumination and illumination‐history effects during β extraction.

**FIGURE 2:**
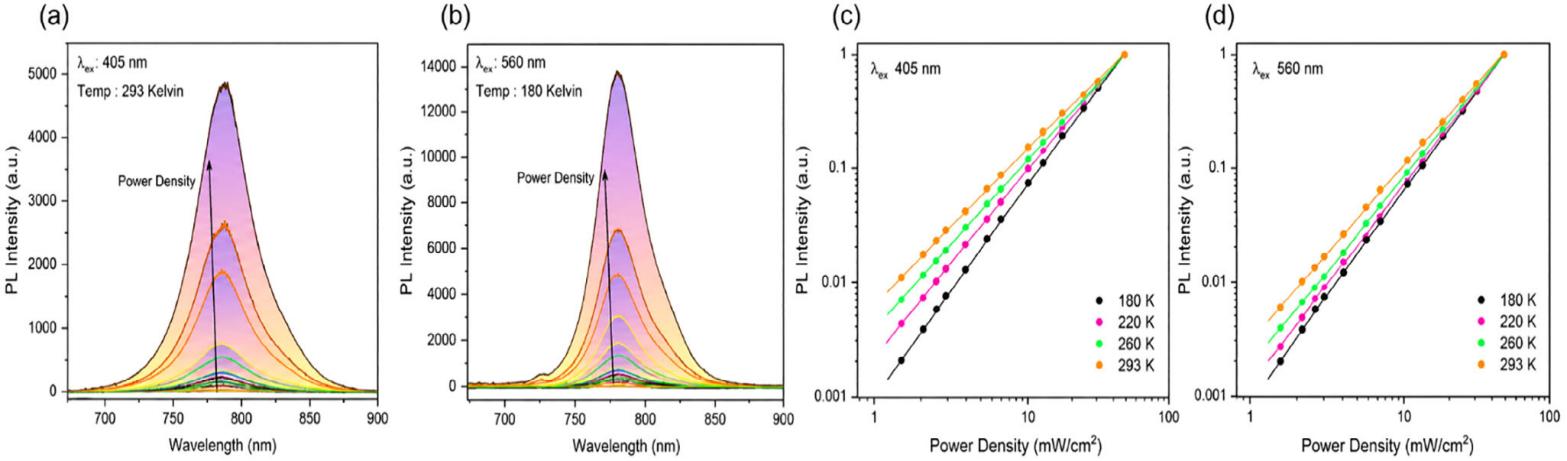
Excitation‐density‐dependent photoluminescence response of MAPbI_3_ thin films under varying spectral and thermal conditions. (a,b) Power‐dependent PL spectra collected under (a) 405 nm excitation at 293 K and (b) 560 nm excitation at 180 K, showing the systematic increase in PL intensity with increasing excitation density. (c,d) Log‐log plots of the normalized maximum PL intensity as a function of excitation density at different temperatures under (c) 405 nm and (d) 560 nm excitation. The extracted slopes reveal persistent intermediate superlinear behavior and its systematic evolution with excitation wavelength and temperature. Each PL spectrum was acquired using a CCD integration time of 0.5 s, maintained throughout each power‐dependent measurement series. Spectra were collected sequentially with an interval of approximately 2 s between successive acquisitions, during which the excitation was blocked to minimize cumulative illumination.

The dependence of β on excitation wavelength indicates that the excitation profile influences not only where carriers are generated, but also the relaxation environment through which they subsequently evolve. As the excitation wavelength increases from 375 to 405 and 560 nm, β increases reproducibly (Figure 3a), following the corresponding increase in optical penetration depth in MAPbI_3_ from approximately 22 nm at 375 to 31 nm at 405 and 67 nm at 560 nm [12, 57]. Short‐wavelength excitation therefore places greater weight on the near‐surface region, where surface‐associated nonradiative loss, local defect populations, and energetic disorder more strongly influence carrier relaxation, whereas longer‐wavelength excitation increasingly samples the film interior (Figure 3b). Longer‐wavelength excitation samples a larger fraction of the film interior and changes the balance among near‐surface loss, trap‐mediated relaxation, carrier redistribution, and bulk radiative recombination. Spatially nonuniform generation therefore defines the initial carrier‐density distribution, while trap filling, carrier redistribution, and surface recombination contribute to the subsequent radiative response. These processes determine the carrier‐population response to the local excitation profile, while in soft halide perovskites the relaxation environment itself can evolve concurrently through carrier‐lattice coupling, dynamic disorder, and anharmonic lattice motion [13, 58, 59]. In soft halide perovskites, where strong carrier‐lattice coupling, dynamic disorder, and anharmonic lattice motion continuously modify the energetic landscape experienced by photoexcited carriers, the apparent PL scaling reflects the coupled evolution of carrier populations and the nonequilibrium relaxation environment in which recombination occurs. The wavelength‐dependent β therefore characterizes an excitation‐conditioned radiative response rather than a simple consequence of the initial carrier‐generation profile.

**FIGURE 3:**
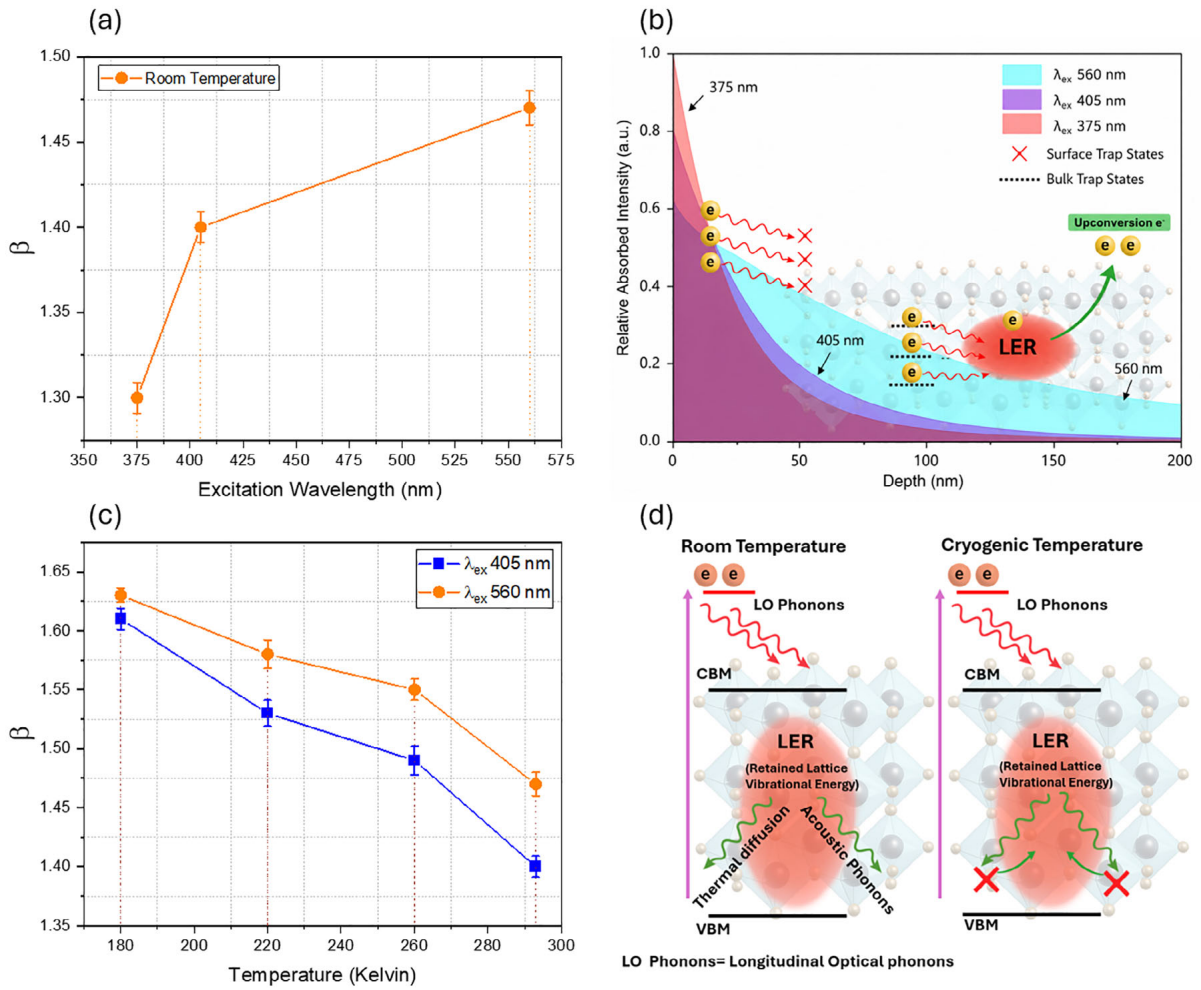
Excitation‐wavelength and temperature dependence of PL superlinearity in MAPbI_3_ thin films. (a) Power‐law exponent (β) extracted from power‐dependent PL measurements as a function of excitation wavelength at room temperature, showing a systematic increase in β with increasing excitation wavelength. (b) Schematic illustration of the depth‐dependent excitation profile under different excitation wavelengths. Short‐wavelength excitation generates carriers predominantly near the illuminated surface, where surface‐associated nonradiative loss, local defect populations, and energetic disorder contribute more strongly to carrier relaxation, whereas longer‐wavelength excitation samples a larger fraction of the film interior. The resulting change in the excitation profile modifies the relative contributions of surface and bulk relaxation pathways and establishes the initial conditions for the subsequent excitation‐dependent radiative response. (c) Temperature dependence of the extracted β values under 405 and 560 nm excitation, showing enhanced superlinear behavior at lower temperature. (d) Conceptual illustration of the influence of thermal conditions on lattice‐energy relaxation within the excited lattice. Carrier‐phonon coupling transfers excitation energy from the electronic system to lattice modes, followed by redistribution among the broader vibrational degrees of freedom of the anharmonic lattice. LO‐phonon‐mediated Fröhlich coupling represents an established carrier‐to‐lattice energy‐transfer pathway, while the subsequent nonequilibrium response involves the broader lattice degrees of freedom. Temperature‐dependent changes in carrier detrapping, vibrational redistribution, and energy dissipation collectively modify the relaxation environment associated with the observed PL response.

The temperature dependence adds a complementary constraint. For both 405 and 560 nm excitation, β increases as temperature decreases from 293 to 180 K (Figure 3c). This trend is compatible with a distributed‐trap recombination picture in which reduced thermal escape at lower temperature prolongs trap occupation and increases the apparent PL slope within SRH‐type statistics [14, 24]. However, cooling also modifies the carrier‐lattice and lattice‐energy relaxation processes accompanying carrier relaxation. In hybrid lead‐halide perovskites, Fröhlich coupling between charge carriers and polar LO phonons represents a major carrier‐phonon interaction near room temperature [34], while energy transferred from photoexcited carriers to optical phonons can subsequently be redistributed through coupled vibrational modes and ultimately dissipated through the lattice. The resulting nonequilibrium response therefore involves broader vibrational and structural degrees of freedom rather than an isolated LO‐phonon population [50, 60]. Temperature consequently modifies both carrier trapping and detrapping and the vibrational redistribution and dissipation accompanying carrier relaxation [32, 33, 50, 60]. The measurements remain above the tetragonal‐to‐orthorhombic transition of MAPbI_3_, reported near 163 K, excluding a low‐temperature phase transition as the primary origin of the observed trend [61]. The increase in β upon cooling is therefore considered within a recombination landscape in which trap‐mediated carrier dynamics and lattice‐energy relaxation evolve concurrently. Reduced thermal escape can strengthen trap‐related contributions to superlinear scaling, while temperature‐dependent changes in vibrational redistribution and dissipation modify the relaxation environment in which trapping, recovery, and radiative recombination occur [14, 24, 32, 33, 50, 60]. The observed thermal evolution is thus consistent with contributions from both carrier‐population processes and temperature‐dependent lattice relaxation (Figure 3d). Together with the wavelength dependence, these results indicate that β reflects an excitation‐conditioned balance among carrier trapping and detrapping, carrier‐lattice energy transfer, vibrational redistribution, and radiative recombination rather than a fixed recombination‐order transition.

### 3.2 Illumination‐History Dependence

Illumination‐history measurements reveal a further dimension of the condition‐dependent PL response. Power‐dependent PL measurements were acquired before and after 10 min of continuous illumination (Figure 4a), with the corresponding spectral evolution provided in Figure S6. The illuminated state exhibits a substantially higher PL intensity across the measured excitation‐density range (Figure 4b), while the fitted exponent decreases from β = 1.42 to β = 1.36. The coexistence of stronger emission and reduced apparent superlinearity is the key feature of this experiment. The PL enhancement shows that prolonged excitation increases the measured radiative output, yet the lower β indicates that this increase is not produced by a simple shift toward a more strongly quadratic excitation dependence. The illumination‐conditioned state instead carries a different balance of excitation‐dependent radiative processes: the absolute PL signal increases, while the apparent scaling moves away from the quadratic limit. This decoupling between PL intensity and β indicates that the exponent cannot be interpreted as a simple monotonic measure of radiative efficiency. The relevant timescale separates this response from the short power‐dependent scans used to extract β. Each β value describes the excitation dependence within a given measurement state, whereas the difference between the initial and illuminated states reflects a slower evolution over minutes of continuous exposure. Such minute‐scale evolution is consistent with illumination‐history effects widely reported in MHPs, including light‐induced photobrightening, trap passivation, changes in defect occupation, ionic redistribution, local structural relaxation, and slow changes in radiative recombination efficiency [17, 23, 42, 54, 62]. These processes need not represent independent relaxation pathways, since changes in defect occupation, ionic configuration, and local lattice structure can evolve concurrently under sustained excitation. The resulting recombination kinetics therefore reflect not only the population of active defect states but also the evolving material environment in which carrier trapping, recovery, and radiative recombination occur. The persistence of illumination‐induced modification is further evident from complementary illumination‐dark‐illumination measurements in FA_0_._8_MA_0_._2_PbI_3_, selected for its pronounced illumination‐history response (Figure S5). Following an initial illumination period, subsequent excitation after 30 and 60 min of dark storage produces PL trajectories that remain dependent on the preceding illumination history under 405, 520, and 620 nm excitation. Both the initial PL level and the normalized temporal evolution differ between successive illumination periods, demonstrating persistence of the photoinduced radiative state on timescales far exceeding the photocarrier lifetime. This long‐lived response indicates involvement of a material degree of freedom that persists beyond carrier recombination, while its microscopic origin may involve coupled evolution of defect occupation, ionic configuration, local structural relaxation, and vibrational energy redistribution.

**FIGURE 4:**
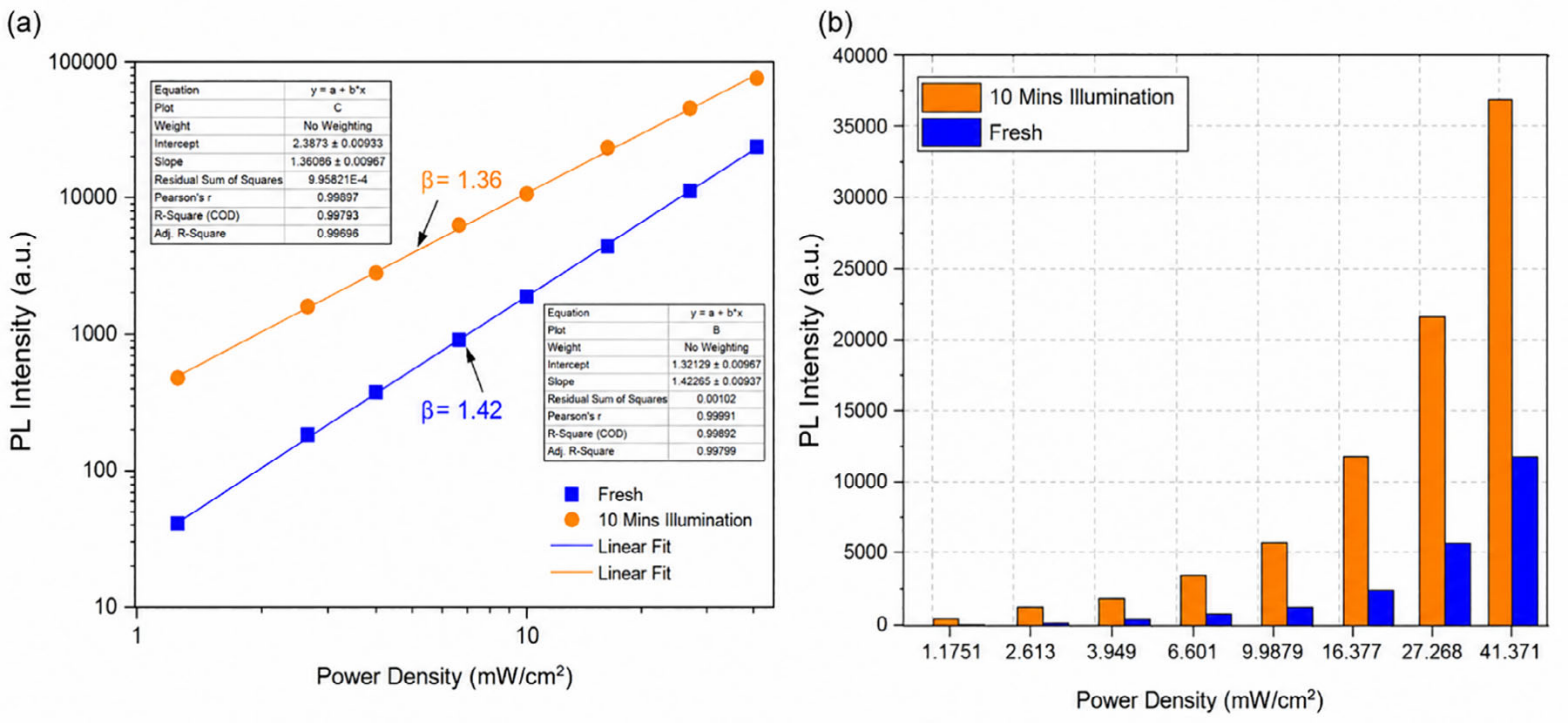
Illumination‐history dependence of photoluminescence superlinearity in MAPbI_3_ thin films. (a) Power‐dependent PL intensity measured before (“Fresh”) and after 10 min of continuous illumination. Sustained illumination produces a pronounced increase in PL intensity across the measured excitation range, accompanied by a reduction in the extracted power‐law exponent β from 1.42 to 1.36. The simultaneous increase in PL intensity and decrease in β shows that radiative output and excitation scaling evolve differently under prolonged illumination. (b) Direct comparison of PL intensities before and after continuous illumination at each excitation power density.

The accompanying spectral evolution provides an additional signature of this illumination‐conditioned state. Under continuous excitation, the progressive increase in MAPbI_3_ PL intensity is accompanied by a small but systematic redshift of the emission maximum, while the spectral linewidth exhibits only modest variation (Figure S6). The correlated evolution of emission intensity and spectral energy is consistent with prolonged illumination modifying the energetic environment from which radiative recombination occurs, rather than producing only a change in emission amplitude. Such spectral evolution may reflect contributions from local structural relaxation, strain, defect occupation and carrier‐lattice interactions and is therefore not assigned to a unique microscopic origin. Instead, its development on the same illumination timescale as the PL evolution provides complementary evidence for a progressively evolving photoexcited relaxation environment. The illumination‐dependent evolution of PL intensity, excitation scaling, and emission energy, combined with the persistence of illumination history after dark storage, supports a picture in which the radiative response develops within an excitation‐conditioned relaxation environment. Carrier trapping, recovery, ionic and structural reorganisation, and radiative recombination can therefore evolve as coupled components of this nonequilibrium material state. Within the proposed framework, β reflects the excitation‐dependent radiative response of this evolving state rather than solely a transition between fixed recombination‐order limits.

### 3.3 Time‐Resolved Photoluminescence Dynamics

Time‐resolved photoluminescence (TRPL) measurements probe the recombination dynamics associated with the condition‐dependent evolution of PL superlinearity observed in the steady‐state response. Decay traces were collected from MAPbI_3_ thin films under varying excitation fluence, temperature, and excitation wavelength (Figure 5a–c and Figure S7). Pulsed 375 nm and 405 nm excitation were used for TRPL measurements, while 560 nm excitation was restricted to steady‐state analysis. The decay profiles were acquired at an excitation repetition rate of approximately 1 MHz using a 256 ps channel width and 3800 temporal channels, corresponding to a 972.8 ns observation window. The ∼1 µs interpulse period and ∼973 ns acquisition window provide sufficient temporal separation and coverage for the longest fitted slow component (τ_2_ ∼ 100 ns). The decay curves were fitted using a biexponential form, $I_{\left(t\right)}=A_{1}e^{-t/\tau_{1}}$ + $A_{2}e^{-t/\tau_{2}}$ where τ_1_ represents the fast decay component, while τ_2_ represents the slower emissive component resolved within the measured temporal window. The amplitude ratio $\frac{A_{2}}{A_{1}}$ provides a measure of the relative contribution of the slower component to the overall PL response.

**FIGURE 5:**
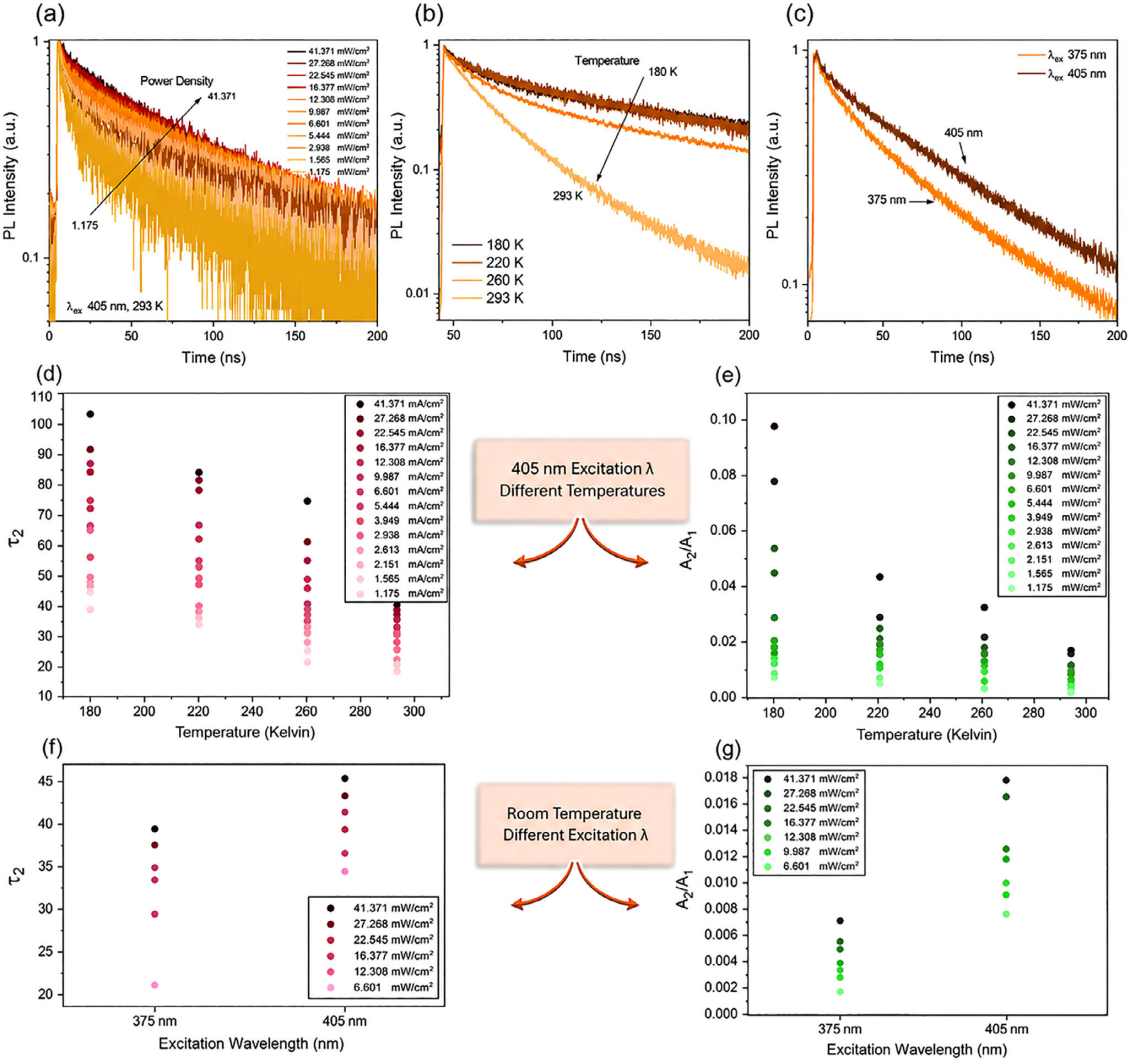
Time‐resolved photoluminescence (TRPL) evolution under varying excitation, thermal, and spectral conditions in MAPbI_3_ thin films. (a) TRPL decay curves collected at 293 K under 405 nm pulsed excitation across a range of excitation power densities (1.175‐41.371 mW cm^−^^2^), showing progressive enhancement of the slower decay component with increasing excitation density. (b) Temperature‐dependent TRPL decay profiles measured under 405 nm excitation, revealing increasingly prolonged decay behavior at lower temperatures. (c) Comparison of TRPL decay traces acquired under 375 nm and 405 nm excitation at room temperature and matched excitation density, showing slower decay under deeper‐penetrating 405 nm excitation. (d) Extracted slow‐component lifetime (τ_2_) as a function of temperature and excitation density under 405 nm excitation. The systematic increase in τ_2_ at lower temperature and higher excitation density indicates strengthening of the delayed emissive contribution. (e) Corresponding amplitude ratio (A_2_/A_1_) extracted from biexponential fitting under 405 nm excitation, showing an increasing relative contribution of the slow component under conditions of reduced vibrational dissipation. (f) Comparison of τ_2_ values extracted under 375 nm and 405 nm excitation at room temperature, showing longer slow‐component lifetimes under 405 nm excitation. (g) Comparison of A_2_/A_1_ values under 375 and 405 nm excitation, indicating a stronger relative contribution of the delayed emissive component under longer‐wavelength excitation. Collectively, the TRPL evolution follows the same excitation‐dependent trends observed in the steady‐state PL superlinearity measurements, linking enhanced delayed emission with the conditions that favor stronger lattice‐conditioned radiative response.

At room temperature under 405 nm excitation, increasing fluence produces a systematic increase in both τ_2_ and $\frac{A_{2}}{A_{1}}$ (Figure 5d,e), indicating that the slower emissive component gains increasing weight at higher excitation density. This behavior differs from the monotonic lifetime shortening commonly associated with increasing bimolecular or Auger‐assisted recombination in semiconductors, where higher carrier density accelerates carrier decay [17, 22, 63, 64, 65]. Instead, the TRPL response develops toward progressively longer‐lived and more strongly weighted delayed emission as excitation density increases. A related evolution is observed in the temperature‐dependent TRPL response. As temperature decreases from 293 to 180 K, both τ_2_ and $\frac{A_{2}}{A_{1}}$ increase systematically (Figure 5d,e), showing that the slower emissive component becomes increasingly pronounced at lower temperature. This trend coincides with reduced phonon‐mediated thermal dissipation in the soft perovskite lattice [32, 33, 66]. The strengthening of the slow component follows the same overall direction as the temperature‐dependent increase in β observed in the steady‐state measurements, consistent with delayed emission becoming more prominent under conditions of reduced thermal relaxation in the excited lattice. The excitation‐wavelength dependence shows the same behavior from a spatial perspective. At matched room‐temperature conditions, 405 nm excitation produces both longer τ_2_ and $\frac{A_{2}}{A_{1}}$ values than 375 nm excitation (Figure 5f,g). Since 405 nm light penetrates deeper into MAPbI_3_ than 375 nm excitation, the slower emissive component becomes increasingly pronounced as carrier generation shifts away from the most surface‐dominated region of the film and toward deeper bulk regions. The temporal response therefore follows the same depth dependence as the steady‐state β evolution, linking deeper excitation with a recombination environment in which delayed radiative relaxation is less strongly suppressed by near‐surface loss pathways.

Across fluence, temperature, and excitation wavelength, the slower TRPL component gains lifetime and relative amplitude under the same conditions that produce stronger intermediate PL superlinearity in the steady‐state measurements. The correlated evolution of β and delayed emission links excitation scaling directly to the temporal evolution of the emissive relaxation landscape. Within the dual‐channel framework, the increasing lifetime and weight of the slow component are consistent with a greater contribution from lattice‐conditioned delayed radiative recovery as the photoexcited relaxation environment evolves. Together with the illumination‐history‐dependent evolution of PL intensity and β, these results establish a coupled response in which excitation scaling, delayed carrier recovery, and radiative efficiency evolve with the state established during excitation. The nonlinear PL response therefore emerges from the interplay among carrier trapping and recovery, carrier‐lattice relaxation, and vibrational‐energy dissipation in the excited soft lattice.

## 4 Lattice Contribution to PL Superlinearity

The correlated evolution of excitation scaling and delayed emission provides a direct experimental link between β and changes in the relaxation dynamics of the photoexcited state. Lower temperature and deeper excitation increase β while simultaneously extending the slow emissive lifetime and increasing its relative contribution, whereas increasing excitation density further strengthens the delayed response. Illumination history modifies this relationship on longer timescales, producing a higher‐emissivity state with a reduced β. These observations show that β evolves together with delayed carrier recovery and with the illumination‐conditioned material state. They do not, however, directly resolve the microscopic lattice configuration responsible for this evolution. Within the framework proposed here, strong carrier‐phonon interactions, anharmonic lattice dynamics, and inefficient vibrational dissipation provide a physical basis for considering a nonequilibrium lattice contribution to this evolving relaxation environment [27, 32, 33, 41, 42, 60]. Trap occupation and defect‐mediated recombination can therefore remain integral to the response, while their kinetics may also be conditioned by the evolving photoexcited environment.

Under these conditions, delayed recovery of weakly localized carriers may become more favorable as the evolving nonequilibrium lattice environment modifies the relaxation conditions governing carrier localization, detrapping, and radiative recovery. Figure 6 summarizes this interpretation. As excitation fluence increases, carrier‐lattice energy transfer and the resulting nonequilibrium vibrational response become increasingly important components of the photoexcited relaxation environment. Energy deposited into the lattice can be redistributed among coupled vibrational and structural degrees of freedom while competing with dissipation to the surrounding material. Within the proposed framework, this evolving environment may alter the balance among carrier localization, recovery, and radiative recombination, consistent with the systematic increase in the lifetime and relative amplitude of the delayed TRPL component. Within the dual‐channel description, changes in the relative weighting of the conventional and lattice‐conditioned radiative contributions allow PL intensity and β to evolve independently, with important implications for the interpretation of PLQY‐based material comparisons. In this regime, β is an effective exponent shaped by carrier relaxation, trapping, lattice‐energy redistribution and dissipation, and delayed radiative recovery rather than a fixed recombination‐order label. The present measurements do not directly resolve the microscopic dimensions or intrinsic relaxation times of individual nonequilibrium lattice regions. Importantly, the long‐lived illumination‐conditioned response is not assigned to the lifetime of individual phonons or to passive thermal confinement. Reported room‐temperature bulk thermal properties of MAPbI_3_ include a thermal conductivity of approximately 0.35 W m^−^^1^ K^−^^1^, a volumetric heat capacity of approximately 1.28 × 10^6^ J m^−^^3^ K^−^^1^, and a corresponding thermal diffusivity of approximately 2.8 × 10^−^^7^ m^2^ s^−^^1^ [67]. Using the bulk diffusivity illustratively, length scales of 10–100 nm correspond to conventional diffusive timescales, *L* ^2^/α, of approximately 4 × 10^−^^10^–4 × 10^−^^8^ s, many orders of magnitude shorter than the illumination‐history response observed here. The relevant long‐lived response is therefore not interpreted as passive nanoscale heat confinement. Within the proposed LER framework, energy transferred to the lattice can instead be redistributed among vibrational and structural degrees of freedom, including elastic potential energy associated with local lattice distortion or deformation. Strain associated with such distorted regions has been proposed to suppress thermal‐energy dissipation across their boundaries [41]. The resulting response is therefore associated with the coupled evolution of vibrational, structural, and electronic degrees of freedom as the photoexcited relaxation environment evolves. The present measurements provide evidence for this evolution through correlated changes in PL scaling, illumination‐history response, delayed emission dynamics, and accompanying spectral evolution.

**FIGURE 6:**
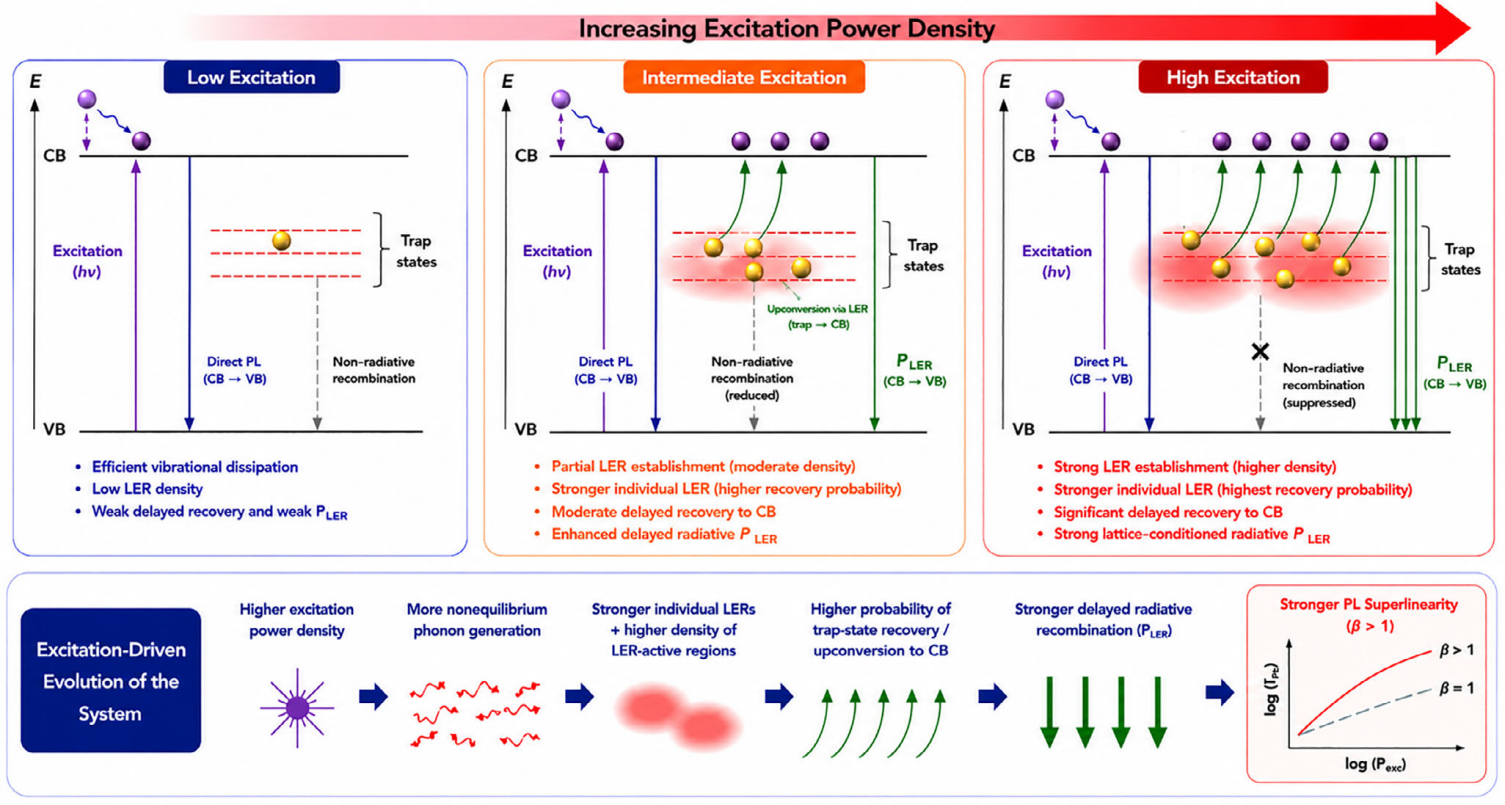
Schematic illustration of the proposed lattice contribution to excitation‐dependent PL superlinearity in MHPs. As excitation fluence increases (left to right), carrier‐phonon coupling transfers increasing excitation energy from the electronic system to the soft perovskite lattice. The deposited energy can subsequently be redistributed among coupled vibrational and structural degrees of freedom while competing with vibrational and thermal dissipation. At low excitation density, conventional trap‐assisted and bimolecular recombination make major contributions to the PL response. At higher excitation density, the balance among carrier‐lattice energy transfer, vibrational redistribution, and energy dissipation establishes a more strongly nonequilibrium relaxation environment. Within the proposed framework, this evolving environment may influence the localization and delayed recovery of weakly localized or shallowly trapped carriers and thereby increase the relative contribution of lattice‐conditioned delayed emission to the overall PL response.

The framework is expected to apply most directly in soft‐lattice hybrid perovskites where free‐carrier recombination retains an effectively quadratic radiative character and vibrational relaxation remains sufficiently slow to sustain nonequilibrium lattice retention. In strongly doped, minority‐carrier‐limited, or exciton‐dominated systems, the balance underlying the present form of PL superlinearity is expected to weaken or disappear [68, 69, 70].

## 5 Conclusion

The present results show that PL superlinearity in MAPbI_3_ evolves with excitation history, excitation profile, temperature, and delayed emission dynamics, supporting an interpretation in which the apparent PL scaling is shaped by an evolving photoexcited relaxation environment in addition to conventional carrier‐population recombination. The correlated evolution of β, delayed emission, and illumination‐history response under varying spectral, thermal, and excitation conditions indicates that the radiative behavior is governed by coupled carrier‐lattice relaxation dynamics. Within the proposed framework, redistribution of excitation energy among vibrational and structural degrees of freedom may introduce a lattice‐conditioned radiative contribution that modifies the apparent PL scaling and redistributes the relative weighting of emissive pathways during excitation. Persistent intermediate β values are therefore consistent with an evolving relaxation environment established in the excited lattice rather than with a single fixed recombination mechanism. These findings provide a physical basis for interpreting excitation‐dependent PL in soft semiconductors beyond static carrier‐population kinetics. More broadly, the results indicate that β, and related PLQY‐based interpretations are excitation‐conditioned observables whose interpretation depends on the evolving relaxation environment established during excitation, rather than being determined solely by fixed recombination‐order assumptions or intrinsic material composition. This perspective motivates a condition‐dependent interpretation of excitation‐dependent PL observables in soft halide perovskites, particularly when using PLQY, β, or lifetime‐derived quantities to evaluate radiative efficiency, defect tolerance, and optoelectronic quality across different materials and measurement regimes.

## Author Contributions

**Mehri Ghasemi**: conceptualization, investigation, writing – original draft, methodology, validation, visualization, writing – review and editing, formal analysis, data curation. **Qi Li**: conceptualization, investigation, methodology, visualization, data curation, writing – review and editing, formal analysis. **Baohua Jia**: funding acquisition, writing – review and editing, project administration, supervision, resources. **Xiaoming Wen**: conceptualization, investigation, funding acquisition, methodology, validation, writing – review and editing, formal analysis, project administration, data curation, supervision, resources.

## Conflicts of Interest

The authors declare no conflicts of interest.

## Supporting information




**Supporting File 1**: advs78378‐sup‐0001‐SuppMat.docx.

**Supporting File 2**: advs78378‐sup‐0002‐SuppMat.docx.

## Data Availability

The data that supports the findings of this study are available in the supplementary material of this article.
