# Supplementary material for: Persistent Photoluminescence Superlinearity Beyond Static Recombination Physics in Metal Halide Perovskites
Source: Adv Sci (Weinh). 2026 Oct 18:e78378. Online ahead of print. doi: 10.1002/advs.78378 (PMC13676996; doi:10.1002/advs.78378)
Supplement: Supplementary file 1 [file ADVS-9999-e78378-s001.docx]

**Persistent Photoluminescence Superlinearity beyond Static Recombination Physics in Metal Halide Perovskites**

Mehri Ghasemi^1&^, Qi Li^1&^, Baohua Jia^1*^, Xiaoming Wen^1*^

^1^School of Science, Centre for Atomaterials and Nanomanufacturing, RMIT University, Melbourne, Victoria 3000, Australia

^&^ These authors have contributed to this work equally

**Photoluminescence measurement**

**1. Steady-state Photoluminescence (PL) Measurement**

Steady-state photoluminescence (PL) measurements were performed using a Zolix DField cooling CCD spectrometer. Excitation sources at 375 nm and 405 nm were provided by Zolix lasers, while the 560 nm excitation light was generated from a Leukos super-continue laser light source filtered through a monochromator to select a central wavelength of 560 nm with a bandwidth of 3 nm.

The CCD integration time for each PL acquisition was fixed at 0.5 s throughout all power-dependent photoluminescence measurements. Neutral density (ND) filters (Thorlabs) were used to adjust the excitation power while maintaining identical acquisition conditions. Spectra were collected sequentially from low to high excitation density with an interval of approximately 2 s between successive acquisitions, during which the excitation laser was blocked to minimise cumulative illumination and illumination-history effects during β extraction.

All spectral data were plotted using Origin 2021.

**2. Time-dependent PL Measurement**

Time-dependent photoluminescence (PL) measurements were conducted using the same Zolix DField cooling CCD spectrometer and excitation sources as described above. The excitation wavelengths used were 405 nm, and 560 nm. For each acquisition, the CCD integration time was adjusted between approximately 0.2 and 0.5 s, depending on the PL intensity. Measurements were recorded at intervals of approximately 3 s over a total duration of 10 minutes. The laser intensity was kept constant throughout the measurement, and all data were collected from the same position on the sample to monitor the evolution of photoluminescence intensity under continuous illumination. All data were collected from the same position on the sample to monitor the evolution of photoluminescence intensity under continuous laser illumination. The laser intensity was kept constant throughout the measurement. All spectral data were plotted using Origin 2021.

**3. Time-resolved Photoluminescence (TRPL)**

Time-resolved photoluminescence (TRPL) measurements were performed using a Zolix Omni-λ300i monochromator equipped with a photomultiplier tube (PMT) UV-Vis detector. Pulsed excitation at 375 nm and 405 nm was used for TRPL measurements, while the 560 nm source was used for steady-state PL measurements only. For the TRPL data presented in the main text, measurements were conducted at an excitation pulse repetition rate of approximately 1 MHz (999995 Hz). The temporal acquisition used a channel width of 256 ps and 3800 channels, corresponding to a total observation window of 972.8 ns. Each TRPL decay trace was accumulated for 60 s to obtain sufficient signal-to-noise for decay analysis. Decay curves were collected from the same sample location under the specified temperature and excitation conditions. Data processing and biexponential decay fitting were carried out using Origin 2021.

**Power Law Fitting for Beta Values**

The power law relationship used to fit the experimental photoluminescence (PL) intensity data is:

$$I_{pl}=AI_{ex}^{\beta}$$

where: $I_{pl}$is the maximum measured PL intensity extracted from each PL spectrum. $I_{ex}$ is the excitation power density. $A$ is a scaling factor related to sample properties. $\beta$ is the power law exponent, which provides insight into the recombination dynamics in the material.

**Fitting Methodology**

1. **Data Preprocessing**

The maximum PL intensity was extracted from each PL spectrum for each excitation wavelength (405 nm, 560 nm, and 375 nm) and temperature (180K, 220K, 260K, and 293K).

A background correction was applied by subtracting the residual intensity at the lowest excitation power to remove baseline contributions.

The data was normalized for more accurate fitting.

2. **Fitting the Power Law**

The direct nonlinear fitting was performed using the equation:

$$I_{pl}=AI_{ex}^{\beta}$$

The parameters 𝐴 and 𝛽 were determined using nonlinear least-squares fitting.

**Log-Log Fit**: Additionally, a logarithmic transformation was applied:

$$\log I_{pl}= \log A+ \beta\log I_{ex}$$

which allowed a linear regression approach for cross-verification.

**Table S1**. Extracted power-law exponents from fitting $I_{pl}=AI_{ex}^{\beta}$, for MAPbI_3_ thin films under different excitation wavelengths and temperatures. A is the pre-exponential factor and β is the power-law exponent.

| **Temperature** | $\beta$ (Wavelength 405 nm) | $\beta$ (Wavelength 560 nm) | $\beta$ (Wavelength 375 nm) |
| --- | --- | --- | --- |
| **180 K** | 1.61±0.009 | 1.63±0.006 |  |
| **220 K** | 1.53±0.011 | 1.58±0.012 |  |
| **260 K** | 1.49±0.012 | 1.55±0.009 |  |
| **293 K** | 1.40±0.009 | 1.47±0.010 | 1.3±0.009 |

**Figure S1**. Steady-state photoluminescence (PL) spectra of MAPbI_3_ thin films under varying excitation power densities at different temperatures and excitation wavelengths. (a) λₑₓ = 375 nm, T = 293 K; (b-e) λₑₓ = 405 nm, T = 180 K, 220 K, 260 K, and 293 K, respectively; (f-i) λₑₓ = 560 nm, T = 180 K, 220 K, 260 K, and 293 K, respectively. Each spectrum corresponds to an incrementally increased excitation power density, revealing a systematic photoluminescence superlinearity trend. The dependence on temperature and excitation wavelength highlights the interplay between optical penetration depth, thermal dissipation, and subgap carrier dynamics.

**Figure S2**. Log-log plots of photoluminescence (PL) intensity versus excitation power density under various excitation wavelengths and temperatures in MAPbI₃ thin films. (a) λₑₓ = 375 nm, T = 293 K; (b-e) λₑₓ = 405 nm, T = 180 K, 220 K, 260 K, and 293 K, respectively; (f-i) λₑₓ = 560 nm, T = 180 K, 220 K, 260 K, and 293 K, respectively. The red lines represent linear fits to the log–log data, yielding the power-law exponent β in each case. Systematic increases in β are observed at lower temperatures and with longer excitation wavelengths, highlighting the nonlinear amplification of PL associated with vibrational energy retention

**Figure S3**. Simulated absorption profiles of excitation light in MAPbI_3_ as a function of depth for three excitation wavelengths (375 nm, 405 nm, and 560 nm), based on wavelength-dependent absorption coefficients.

**Figure S4**. Steady-state photoluminescence (PL) spectra of MAPbI_3_ thin films recorded at varying excitation power densities (1.175 to 41.371 mW/cm²) under 405 nm excitation, comparing (a) the fresh sample and (b) the same region after 10 minutes of continuous illumination. The post-illumination spectra exhibit a substantial increase in PL intensity across all fluences, reflecting enhanced radiative efficiency. This illumination-induced gain is consistent with an illumination-conditioned radiative response in which lattice-mediated delayed recovery contributes to the enhanced PL output.

**Figure S5. Persistent illumination-history dependence in FA₀.₈MA₀.₂PbI₃. Time-dependent PL measured under (a,b) 405 nm, (c,d) 520 nm, and (e,f) 620 nm excitation during three successive illumination periods. Following the initial 12 min illumination period, the sample was stored in the dark for 30 min before the second illumination cycle and subsequently for 60 min before the third cycle. Panels (a,c,e) show the absolute PL evolution during the three illumination periods, while panels (b,d,f) show each illumination cycle normalized to its initial intensity. The PL response after dark storage remains dependent on the preceding illumination history, as reflected in both the initial emission level and subsequent photobrightening kinetics. The persistence of this modified radiative response after 30-60 min in the dark demonstrates a long-lived illumination-conditioned state in this related iodide-perovskite composition.**

**Figure S6. Time-dependent spectral evolution of MAPbI₃ under continuous illumination. (a,e) Time-resolved PL spectral maps for two independently measured MAPbI₃ datasets. (b,f) Integrated PL intensity, (c,g) PL peak position, and (d,h) spectral FWHM as a function of illumination time. Progressive photobrightening is accompanied by a small but reproducible redshift of the PL maximum of approximately 0.6-0.7 nm over 300 s, whereas the spectral linewidth exhibits comparatively modest variation. The spectral shift is not assigned to a unique microscopic mechanism but shows that the illumination-induced PL evolution is accompanied by a measurable change in emission energy.**

**Figure S7**. Time-resolved photoluminescence (TRPL) decay profiles of MAPbI_3_ thin films under varying excitation power densities and thermal conditions. (a) TRPL curves recorded at room temperature (293 K) using a 375 nm pulsed laser. (b-e) TRPL measurements at 180 K, 220 K, 260 K, and 293 K, respectively, under 405 nm excitation, showing progressively longer decay tails with decreasing temperature and increasing fluence. (f) Raw PL decay (orange) and bi-exponential fit (black) for 405 nm excitation at 180 K and 41.371 mW/cm², demonstrating the quality of the kinetic model fit. All decays are normalized in (a-e) to highlight differences in lifetime dynamics across conditions. The observed elongation of the slow decay component (τ₂) and rising amplitude ratio (A₂/A₁) at higher fluences and lower temperatures are consistent with a stronger delayed radiative contribution under conditions that favour reduced vibrational dissipation.
