# Supplementary material for: Persistent Photoluminescence Superlinearity Beyond Static Recombination Physics in Metal Halide Perovskites
Source: Adv Sci (Weinh). 2026 Oct 18:e78378. Online ahead of print. doi: 10.1002/advs.78378 (PMC13676996; doi:10.1002/advs.78378)
Supplement: Supplementary file 2 [file ADVS-9999-e78378-s002.docx]

**Supplementary Information for lattice energy reservoir (LER), dynamic processes, physical effects and published evidence**

The **Lattice Energy Reservoir (LER)** framework is proposed to explain a broad range of **anomalous electronic and optical phenomena** observed in metal halide perovskites (MHPs), which are difficult to reconcile within conventional semiconductor models.

Unlike traditional approaches that treat each phenomenon independently (e.g., long carrier lifetime, photobrightening, or memory effects), the LER theory introduces a **unified physical picture** based on a **closed energy cycle**: **Phonon energy injection → energy storage in the lattice → delayed energy feedback to the electronic system.** This energy cycle provides a consistent explanation for dynamic processes spanning **ultrafast (fs) to ultraslow (seconds–hours) timescales**.

**S1. Lattice Energy Reservoir in Metal Halide Perovskites**

The **Lattice Energy Reservoir (LER)** refers to **localized, dynamically formed nanodomains** within the soft lattice of MHPs that can **store and retain energy**.

**Key physical picture**

- The lattice is not a passive heat sink
- Instead, it can **temporarily store energy** and **release it later**
- This stored energy can **feed back into carrier dynamics**

**Why this is unique in MHPs:** MHPs possess:

- Soft, deformable lattices
- Strong electron–phonon coupling
- Low thermal conductivity

These properties allow:

- **Efficient energy injection into the lattice**
- **Suppressed energy dissipation**
- **Formation of localized energy storage regions (LERs)**

The LER introduces a **three-stage dynamic energy cycle**: illustrated schematically in Figures 1–3

1. **Hot LER formation** (energy storage) **Figure 1**.
2. **Phonon upconversion within LER** (energy redistribution) **Figure 2**.
3. **Subgap carrier upconversion** (energy feedback to electrons) **Figure 3**

**S2. Hot LER Formation (Energy Storage Stage)**

1. **Carrier relaxation**
   - Photoexcited carriers lose excess energy by emitting phonons
   - Strong electron–phonon coupling leads to **polaron formation**
2. **Phonon injection into lattice**
   - Optical phonons decay into acoustic phonons
   - Energy is transferred into lattice vibrations
3. **Energy localization**
   - Due to lattice softness, energy is not uniformly dissipated
   - Instead, it becomes **localized in nanoscale regions**
4. **Elastic energy storage**
   - Local lattice deformation stores energy as **elastic potential energy**

**Figure 1** hot LER formation by phonon injection and accumulation, storage as elastic lattice potential energy.

**Hot LER Formation**

Electron-phonon interaction leads to strong polaron formation in MHPs,^1, 2^ where excess energy from hot carriers rapidly relaxes through hot phonon emissions. The hot electrons then thermalize to the conduction band, primarily via symmetric Klemens decay and anti-symmetric Ridley decay.^3^ The unique soft lattice of MHPs enables efficient phonon-lattice coupling and localized energy storage.^4-6^ As phonon energy is gradually injected, elastic lattice deformation can develop within the LER, leading to increased potential energy relative to the surrounding lattice. Simultaneously, the LER boundary forms a strained interface that suppresses acoustic phonon transport, effectively reducing thermal dissipation. The transport of acoustic phonon can be significantly suppressed therefore the thermal energy dissipation is decreased and high-density longitudinal-acoustic (LA) phonons can be accumulated in LER with higher potential energy over the surrounding. The low energy dissipation can be attributed to strong reflection of acoustic phonons at strain boundaries, which act as phonon cavities,^7^ suppressing thermal transport and thereby extending energy retention times up to seconds or hours. Under continuous illumination or higher-fluence excitation, more phonon energy is stored within LERs as hot LERs, leading to an increased density of hot LERs in the lattice. This dynamic process can be approximately described by a saturation process as observed by time-dependent PL spectroscopy.^8, 9^

**Key mechanism: phonon confinement**

The boundary of an LER is **strained**, which leads to Reflection of acoustic phonons, Suppression of phonon transport, reduced thermal dissipation, this effectively creates a **“phonon cavity”**, where energy is trapped.

**Result:**

- Accumulation of **high-density acoustic phonons (LA phonons)**
- Formation of **“hot LERs”** under continuous illumination
- Energy retention times extended to **milliseconds–hours**

**Figure 2**. LA to LO phonon upconversion.

**S3. Acoustic–Optical Phonon Upconversion**

**Conventional picture** in conventional semiconductors:

- LO phonons decay into LA phonons
- Energy dissipates quickly into the lattice

**LER-modified picture**

Within a hot LER:

- LA phonons accumulate to **very high density**
- Reverse processes become possible: **Acoustic phonons can upconvert into optical phonons**

**Why this is important**

- This process is normally negligible
- It becomes significant only under **extreme phonon density**, which is enabled by LER

**Physical consequence:** creates a **phonon bottleneck**, not due to mismatch, but due to: **phonon accumulation and reverse energy flow**

**Acoustic-Optical Phonon Upconversion**

Upon hot carrier photogenerated, the thermalization generally includes three distinct relaxation stages: (1) hot carrier thermalization (Fröhlich interaction) via carrier-carrier scattering, the hot carriers reach a quasi-equilibrium with the Fermi-Dirac distribution in the timescale of 100 fs. (2) longitudinal-optical (LO) phonons decay to LA phonons by the symmetric Klemens-decay and anti-symmetric Ridley-decay^3^; where one LO phonon decays into two LA phonons with energy and momentum conservations, **Figure 2**. (3) Acoustic phonons propagate and dissipate their energy into the surrounding lattice. Mismatch between LO and LA phonons can result in a phonon bottleneck, slowing the cooling of hot carriers by breaking energy and momentum conservation.^3, 10^ However, it has been confirmed that MHPs do not have such phonon mismatch. Instead, the phonon bottleneck in MHPs is ascribed to abnormal LA-LO phonon upconversion.^11^ We note, acoustic–optical phonon upconversion cannot be achieved unless an extremely high density of LA phonons is present. Therefore, such phonon upconversion as phonon bottleneck is not possible to be observed in normal condition; but it has been widely observed in MHPs at higher density excitation, where hot LERs is the critical necessary condition.

**Figure 3** subgap electron upconversion by multi-phonon absorption.

**S4. Subgap Carrier Upconversion (Energy Feedback Stage)**

**Core idea**

The energy stored in LER is **not lost**—it can be **returned to the electronic system**.

**Mechanism**

- High-density phonons inside LER enable:

**Multi-phonon absorption by subgap electrons**

- Electrons below the conduction band can be:

**re-excited (upconverted) into the conduction band**

**This is unusual**

Under normal conditions:

- Thermal energy (~26 meV at room temperature) is insufficient
- Multi-phonon processes are extremely unlikely

**In LER**

- High phonon density dramatically increases probability
- Makes otherwise negligible processes **physically relevant**

At room temperature, the available thermal energy is only ~26 meV, while the phonon energies in MHPs are typically less than 10 meV.^12, 13^ Anti-Stokes fluorescence for energy gap of two hundreds of meV is highly impossible **in the lattice with uniform potential distribution.** In contrast, in hot LERs the high density of LA phonons makes it rationale.

**Key consequence**

The lattice becomes an **active energy source**, not just a sink

This establishes an energy **feedback loop**: photon 🡺 phonon 🡺 Lattice 🡺 carriers

**S5. Suppressed Energy Dissipation and Ultraslow Dynamics**

**Energy dissipation in conventional systems**

- Energy rapidly spreads across the lattice
- System reaches equilibrium quickly

**In LER systems**

Due to phonon confinement:

- Thermal transport is strongly suppressed
- Energy release becomes **slow and gradual**

**Observable consequences**

This leads to a range of **ultraslow phenomena**:

- Photobrightening (PL enhancement over time)
- Persistent photoconductivity
- Memory effects
- Long-lived lattice polarization
- Illumination-induced transparency

**All these effects originate from: Delayed release of stored lattice energy, rather than purely electronic or defect-related mechanisms.**

**S6. LER-Governed Carrier Dynamics**

With LER, carrier dynamics are fundamentally modified.

**Conventional assumption:** Carrier recombination depends only on instantaneous carrier density

**LER framework:** Carrier dynamics depend on both carrier density and **stored lattice energy,** the system becomes **non-Markovian (history-dependent)**

- Time-dependent recombination rates
- Apparent lifetime extension
- Illumination-history-dependent behavior

**Figure 4.** LER governed electron dynamics. With LERs, a unique energy recycle of storage-feedback is established between electrons and LERs through phonon coupling.

***In simple terms, LER means the lattice can temporarily store energy like a battery and later return it to electrons, which changes how the material responds to light over time.***

**Published evidence for supporting lattice energy reservoir (LER) theory**

Over the past decade, metal halide perovskites (MHPs) have been intensively studied. The anomalous electron dynamics—spanning timescales from femtoseconds to hours—directly contradict current semiconductor theory.

The lattice in MHPs acts as a **dynamic energy reservoir** that can store, retain, and release energy, thereby actively governing electronic processes. LER theory provides a **unified explanation** for many seemingly unrelated experimental observations.

**Figure 5**. LER induced unique physical effects and anomalous phenomena in MHPs.

**LER theory fully explains anomalous phenomena in MHPs,** including:

1. **Slowed cooling of hot carriers,** ascribed to unique phonon upconversion (LA🡺LO) and exclude the phonon bottleneck due to mismatch of LO-LA.
2. **Dynamic long carrier lifetime, defect tolerance, single photon upconversion without doping**, ascribed to unique subgap carrier upconversion occurring exclusively within LERs.
3. **Dynamic PL efficiency, dynamic carrier lifetime** as observed as dynamic defect tolerance, illumination induced PL enhancement, defect curing, defect healing, due to gradually formation under illumination.
4. **Memory effects and persistent effects**, also including memory of PL efficiency, memory of carrier lifetime, ascribed to suppressed energy dissipation by strained LER boundary.
5. **Mobile ion relevant effects:** higher LER potential provides significantly higher probability of ionization, so that mobile ions can be generated even at mild illumination. This is the fundamental mechanism for mixed electron-ionic conduction, degradation and instability.
1. **Slowed cooling of hot carriers, phonon bottleneck by optical-acoustic phonon upconversion**

Significantly slowed cooling of hot carriers has been observed at high density of photogenerated carriers, which is ascribed to acoustic-optical phonon upconversion.**^11, 14-18^**

**Figure S1** (**a**) 3D TA spectrum pumped at 400 nm with an initial carrier concentration *N*_0_=4.84 × 10^18^ cm^−3^. The initial broadening of the bleaching signal extending above the apparent band gap energy indicates the temporary presence of hot carriers. (**b**) Normalized negative TA spectra (−*ΔOD*) at different pump-probe time delays *t* from 0.1 to about 900 ps. (**c**) Decay of the bleaching signal given as minus milli *ΔOD* (−m*ΔOD*) at different wavelengths showing carrier relaxation kinetics.

**Figure S2** (**a**) Carrier temperature-dependent phonon emission lifetime in different lead-halide perovskites with a similar initial carrier concentration of around 2 × 10^18^ cm^−3^. The error bars shows the standard error of the average (s.e.m) emission lifetime. An emission lifetime that is about 10 times longer than the other materials is observed in FAPbI_3_ with carrier temperature at and below 400 K; (**b**) Proposed phonon dynamics in the FAPbI_3_. The solid black line shows the total phonon DOS, in which the contributions from the inorganic and organic sub-lattices are shown by the blue region on the bottom with the pink region stacked on top, respectively. The labelled phonon dynamic process are: (1) Fröhlich interaction of carriers primarily on the lead-halide framework; (2) relaxation of lead-halide LO phonon, organic sub-lattice can be excited by phonon–phonon scattering; (3) propagation of acoustic phonon is blocked due to anharmonic phonon–phonon scatterings; (4) up-conversion of acoustic phonons; and (5) carrier reheating.

Acoustic-optical phonon up-conversion and hot-phonon bottleneck in lead-halide perovskites, [***Nature Communications***](https://www.nature.com/ncomms) **8, 14120 (2017)**

**Figure S3**. Ultrafast hot carrier cooling in LHP NCs. (a, d) Pseudocolor transient absorption (TA) spectroscopies of CsPbBr_3_ NCs and CsPbBr_3_@Cs_4_PbBr_6_ nanocomposites at *n*_0_ ∼ 2.4 × 10^17^ cm^–3^ and 3.6 × 10^18^ cm^–3^ (⟨*N*_0_⟩ ∼ 0.2 and 3). (b, e) The normalized spectral distribution of both samples at different delay times, where *n*_0_*∼* 3.6 × 10^18^ cm^–3^ (⟨*N*_0_⟩ ∼ 3). The shaded areas mark the data used for extracting the electronic temperature. (c, f) Electronic temperature of HCs as a function of delay time for both samples under different excitation fluences.

***Nano Lett.* 2020, 20, 6, 4610–4617 ^19^**

**Figure S4**. Hot-carrier temperature as a function of delay time for the medium MAPbBr_3_ nanocrystals and bulk film with different carrier densities.

[***Nature Communications***](https://www.nature.com/ncomms) **8, 14350 (2017)**

**2. Photogeneration carrier recombinaiton: long and variable carrier lifetime, dynamic range dependent carrier lifetime^20-26^, defect tolerance, single photon upconversion without doping**

Reported carrier lifeitmes of tri-cation perovksites span from 10^-7^-10^-5^ s. The carrier lifetime is found to be relevant to experimental conditions (excitation wavelength and fluence, measurement during, system performance). Therefore, carrier lifetime in MHPs cannot use for evaluating fabrication quality.

**Figure S5. a**, The voltage difference *E*_g_/*q* – *V*_oc_ as a function of decay time of perovskite films. **b**, Meta-analysis on the dynamic range of tr-PL decay curves in publications. Carrier lifetimes are dependent dynamic range of the detection system.

**Figure S6** very long carrier lifetime at a system with very high dynamic range**.**

*Shallow defects and variable photoluminescence decay times up to 280 µs in triple-cation perovskites,* [***Nature Materials***](https://www.nature.com/nmat) ***23, 391–397 (2024)***

3. **Dynamic PL efficiency, dynamic carrier lifetime** as observed as dynamic defect tolerance,

**Figure S7.**  A series of time-resolved PL decays from a MAPbI_3_ film measured over time under illumination. MHPs exhibit various illumination dynamic PL efficiency and dynamic carrier lifetimes.

Positive and Negative Effects under Light Illumination in Halide Perovskites, **Small Science**, 4 (2024) 2400028

**Figure S8**. Illuminaiton induced PL enhancement in MAPbI3 film. PL intensities over time under (a) continuous excitations at 0.2 and 0.65 W/cm^2^, (b) apparent memory effect, when sample keeps in dark for 200 s, PL efficiency remind approxiamtely not decrease.

**Solar RRL 1 (2017) 1600001**

**Photobrightening effect cmparison between perovskite (MAPbI_3_) and conventional semiconductor (Sb_2_Se_3_):** perovskite exhibits significant photobrightening

**Samples with similar structure in MAPbI_3_ and Sb_2_Se_3_**

**Figure S9** Surface photovoltage voltage **(**SPV) spectra of (a) Sb_2_Se_3_ and (b) MAPbI_3_ perovskite film under different durations of AM 1.5G simulated sunlight exposure, where L- represents the light exposure time.

Bian et al. Deciphering of light-soaking behavior in perovskite: Electro-optic analysis of intrinsic MAPbI3 versus Sb2Se3, **J. Photochem. & Photobiology A: Chem.** 2026 Pages 116970

**Figure S10** contact potential difference (CPD) as a function of illumination time (min)

Bian et al. Deciphering of light-soaking behavior in perovskite: Electro-optic analysis of intrinsic MAPbI3 versus Sb2Se3, **J. Photochem. & Photobiology A: Chem.** 2026 Pages 116970

3. **Highly efficient anti-Stokes single photon upconversion^27, 28^ with energy gap up to 220 meV^27, 29, 30^.** Efficent anti-Stokes PL are independently observed in many groups in various perovksites.

**Figure S11**. Schematic diagram for Anti-Stokes upcovnersion PL in perovskite.

***Nano Research 13 (2020) 1962***

**Figure S12** (a) linear absoprtion and PL spectrum and excitation energy, (b) Upconversion PL as a fucntion of excitation energy, power and temperatures.

***Nano Research 13 (2020) 1962***

**Figure S13**. (A) Energy level schematic of ASPL mechanism illustrating how different excitation energy leads to different up-conversion energy gains, Δ*E*. (B) Arrhenius plots of the thermal activation of ASPL intensity with different Δ*E*s. The blue, green, yellow, and orange traces represent data where Δ*E* is 28, 37, 50, and 72 meV, respectively. (C) The activation energy, *E*_a_, versus the up-conversion energy gain, Δ*E*, as shown in (B). The data points are shown in blue, with line of best fit shown in dashed blue. The gray dashed line represents the hypothetical scenario where *E*_a_ = Δ*E*.

**ACS Nano 2024, 18, 28, 18457**

**Figure S14**. The DC and UC PL properties of PEPI micro platelets. (C) Absorption, PLE, and excitation energy–dependent PL spectra of a PEPI micro platelet.(**D**) Excitation power dependence of the PL intensity for a PEPI micro platelet. (**E**) Temperature dependence of the UC and DC PL intensity and bandwidth.

**Science Advances 2023, Vol 9,** [**DOI: 10.1126/sciadv.adi934**](https://doi.org/10.1126/sciadv.adi9347)

**Figure S15** Schematics of the energy diagram and dynamics of UC in perovskites.

**Science Advances 2023, Vol 9,** [**DOI: 10.1126/sciadv.adi934**](https://doi.org/10.1126/sciadv.adi9347)

**Figure S16** (a) The schematic of the LER-induced upconversion in space regime. (b) The energy diagram of the LER upconversion process.

Lu et al. **Materials Today** 86 (2025) 340-355 <https://doi.org/10.1016/j.mattod.2025.03.008>

4. **Mobile ion induced PL quenching under mild illumination**

**Figure S16** (a) Schematic experimental setup, b) optical image without voltage, and c,d) images with voltage of 10 V (0.5 v/μm) in 200 and 400 s.

Wen et al. **Advanced Materials Interfaces** 2016, 3, 1600467

**Figure S17** PL intensities over time under continuous excitation with various intensities for a) perovskite, inset: setup for time dependent PL measurement; b) perovskite/PCBM, c) perovskite/spiro samples. (Solid curves are dual exponential fits.). d) Time constants extracted from c) using dual exponential fitting plotted on double logarithmic scales.

Wen et al. **Advanced Materials Interfaces** 2016, 3, 1600467

**Figure S18** Schematic diagrams of dynamic processes occurring at the perovskite/spiro interface: a) photogenerated holes transfer into spiro and an internal electric filed establishes in nanosecond timescale; b) negative ions migrate and accumulate at the interface; c) after switching off the illumination, holes relax quickly and ions drift slowly toward the lower density region

Wen et al. **Advanced Materials Interfaces** 2016, 3, 1600467

**Significant subgap absorption** (important for subgap upconversion!)

Rich subgap absorption has been found in MHPs.

**Figure S19** Schematic depiction of charge carrier recombination via tail states, including charge trapping-detrapping, nonradiative recombination.

**Adv Fun Mater. 28, 2018, 1801808**

Due to significant subgap absorption, the different bandgap is apparently shown in different thickness, we demonstrate this error is ascribed to unique subgap absorption of MHPs, the thickness of single crystals, and the technical limitation of the spectrophotometer.

**Figure S20** (a) Calculated absorption of MAPbI_3_ films with different thicknesses. (b-c) Absorption spectra of MAPbI_3_ thin films and single crystals with different thicknesses.

**Phys. Chem. Chem. Phys., 2024, 26, 9137**

**Figure S21**. Sub-bandgap EQE of four p–i–n perovskite solar cells using an ITO back electrode. a Without optical spacer or mirror. b With an MgF_2_ optical spacer and an Ag mirror. The thickness of the MgF_2_ layers was 100, 150, 200, and 300 nm as indicated in the legend.

**Nature Communications** **13**, 349 (2022)

5. **Persistent structure polarization^31^, memory effect^32, 33^**

**Figure S22**. Long-lasting persistent photoconductance that shows no apparent sign of decay during the measurement timescale (**a**), and expanded version of the initial 2,000 s, with the arrows highlighting the light-on and light-off events (**b**). The blue and grey shaded areas correspond to the light-on and -off regions, respectively. **c**, Persistent photoconductance at 200 K compared with that at 10 K, where the conductance *G* is normalized by the conductance under illumination *G*_light_. The persistent photocarrier lifetime *τ* at 10 K and 200 K is 1.0 × 10^6^ s and 7.8 × 10^2^ s, respectively.

[***Nature Nanotechnology***](https://www.nature.com/nnano) **18, 357–364 (2023)**

**Figure S23**. Resistive switching performance of CH_3_NH_3_PbI_3_-based flexible nonvolatile memory device. (a) *I*–*V* characteristics of the Au/perovskite/ITO structure. The insets show photographs of fully fabricated memory devices on a flexible substrate and top electrode configuration of memory devices (scale bar: 300 μm). (b) Data retention characteristics of LRS and HRS states. (c) Switching endurance of the flexible perovskite memory device.

***ACS Nano* 2016, 10, 5, 5413–5418**

**References:**

(1) Guzelturk, B.; Winkler, T.; Van de Goor, T. W.; Smith, M. D.; Bourelle, S. A.; Feldmann, S.; Trigo, M.; Teitelbaum, S. W.; Steinrück, H.-G.; de la Pena, G. A. Visualization of dynamic polaronic strain fields in hybrid lead halide perovskites. *Nat. Mater.* **2021**, *20* (5), 618-623.

(2) Meggiolaro, D.; Ambrosio, F.; Mosconi, E.; Mahata, A.; De Angelis, F. Polarons in metal halide perovskites. *Adv. Energy Mater.* **2020**, *10* (13), 1902748.

(3) Conibeer, G.; König, D.; Green, M.; Guillemoles, J. Slowing of carrier cooling in hot carrier solar cells. *Thin Solid Films* **2008**, *516* (20), 6948-6953.

(4) Wang, L.; Liu, P.; Guan, P.; Yang, M.; Sun, J.; Cheng, Y.; Hirata, A.; Zhang, Z.; Ma, E.; Chen, M.; et al. In situ atomic-scale observation of continuous and reversible lattice deformation beyond the elastic limit. *Nat. Comm.* **2013**, *4* (1), 2413. DOI: 10.1038/ncomms3413.

(5) Guo, Z.; Wang, J.; Yin, W.-J. Atomistic origin of lattice softness and its impact on structural and carrier dynamics in three dimensional perovskites. *Energy Enviro. Sci.* **2022**, *15* (2), 660-671.

(6) Jin, B.; Cao, J.; Yuan, R.; Cai, B.; Wu, C.; Zheng, X. Strain relaxation for perovskite lattice reconfiguration. *Advanced Energy and Sustainability Research* **2023**, *4* (4), 2200143.

(7) Trigo, M.; Bruchhausen, A.; Fainstein, A.; Jusserand, B.; Thierry-Mieg, V. Confinement of Acoustical Vibrations in a Semiconductor Planar Phonon Cavity. *Phys. Rev. Lett.* **2002**, *89* (22), 227402. DOI: 10.1103/PhysRevLett.89.227402.

(8) Andaji‐Garmaroudi, Z.; Anaya, M.; Pearson, A. J.; Stranks, S. D. Photobrightening in Lead Halide Perovskites: Observations, Mechanisms, and Future Potential. *Adv. Energy Mater.* **2020**, *10* (13), 1903109.

(9) Chen, S.; Wen, X.; Huang, S.; Huang, F.; Cheng, Y. B.; Green, M.; Ho‐Baillie, A. Light illumination induced photoluminescence enhancement and quenching in lead halide perovskite. *Solar RRL* **2017**, *1* (1), 1600001.

(10) Pandey, A.; Guyot-Sionnest, P. Slow electron cooling in colloidal quantum dots. *Science* **2008**, *322* (5903), 929-932.

(11) Yang, J.; Wen, X.; Xia, H.; Sheng, R.; Ma, Q.; Kim, J.; Tapping, P.; Harada, T.; Kee, T. W.; Huang, F. Acoustic-optical phonon up-conversion and hot-phonon bottleneck in lead-halide perovskites. *Nat. Comm.* **2017**, *8*, 14120.

(12) Chu, W.; Zheng, Q.; Prezhdo, O. V.; Zhao, J.; Saidi, W. A. Low-frequency lattice phonons in halide perovskites explain high defect tolerance toward electron-hole recombination. *Science advances* **2020**, *6* (7), eaaw7453.

(13) Zhao, D.; Hu, H.; Haselsberger, R.; Marcus, R. A.; Michel-Beyerle, M.-E.; Lam, Y. M.; Zhu, J.-X.; La-o-vorakiat, C.; Beard, M. C.; Chia, E. E. Monitoring Electron–Phonon Interactions in Lead Halide Perovskites Using Time-Resolved THz Spectroscopy. *ACS Nano* **2019**, *13* (8), 8826-8835.

(14) Lin, W.; Canton, S. E.; Zheng, K.; Pullerits, T. Carrier Cooling in Lead Halide Perovskites: A Perspective on Hot Carrier Solar Cells. *ACS Energy Lett.* **2023**, *9* (1), 298-307.

(15) Li, M.; Fu, J.; Xu, Q.; Sum, T. C. Slow Hot‐Carrier Cooling in Halide Perovskites: Prospects for Hot‐Carrier Solar Cells. *Adv. Mater.* **2019**, *31* (47), 1802486.

(16) Joshi, P. P.; Maehrlein, S. F.; Zhu, X. Dynamic screening and slow cooling of hot carriers in lead halide perovskites. *Adv. Mater.* **2019**, *31* (47), 1803054.

(17) Shen, Q.; Ripolles, T. S.; Even, J.; Ogomi, Y.; Nishinaka, K.; Izuishi, T.; Nakazawa, N.; Zhang, Y.; Ding, C.; Liu, F. Slow hot carrier cooling in cesium lead iodide perovskites. *Appl. Phys. Lett.* **2017**, *111* (15), 153903.

(18) Fu, J.; Xu, Q.; Han, G.; Wu, B.; Huan, C. H. A.; Leek, M. L.; Sum, T. C. Hot carrier cooling mechanisms in halide perovskites. *Nat. Comm.* **2017**, *8* (1), 1300. DOI: 10.1038/s41467-017-01360-3.

(19) Nie, Z.; Gao, X.; Ren, Y.; Xia, S.; Wang, Y.; Shi, Y.; Zhao, J.; Wang, Y. Harnessing hot phonon bottleneck in metal halide perovskite nanocrystals via interfacial electron–phonon coupling. *Nano Lett.* **2020**, *20* (6), 4610-4617.

(20) deQuilettes, D. W.; Frohna, K.; Emin, D.; Kirchartz, T.; Bulovic, V.; Ginger, D. S.; Stranks, S. D. Charge-Carrier Recombination in Halide Perovskites: Focus Review. *Chem. Rev.* **2019**, *119* (20), 11007-11019.

(21) Wen, X.; Feng, Y.; Huang, S.; Huang, F.; Cheng, Y.-B.; Green, M.; Ho-Baillie, A. Defect trapping states and charge carrier recombination in organic–inorganic halide perovskites. *J. Mater. Chem. C* **2016**, *4* (4), 793-800.

(22) Leijtens, T.; Eperon, G. E.; Barker, A. J.; Grancini, G.; Zhang, W.; Ball, J. M.; Kandada, A. R. S.; Snaith, H. J.; Petrozza, A. Carrier trapping and recombination: the role of defect physics in enhancing the open circuit voltage of metal halide perovskite solar cells. *Energy Enviro. Sci.* **2016**, *9* (11), 3472-3481.

(23) Stranks, S. D.; Burlakov, V. M.; Leijtens, T.; Ball, J. M.; Goriely, A.; Snaith, H. J. Recombination Kinetics in Organic-Inorganic Perovskites: Excitons, Free Charge, and Subgap States. *Phys Rev Appl* **2014**, *2* (3), 034007.

(24) Dong, Q.; Fang, Y.; Shao, Y.; Mulligan, P.; Qiu, J.; Cao, L.; Huang, J. Electron-hole diffusion lengths> 175 μm in solution-grown CH3NH3PbI3 single crystals. *Science* **2015**, *347* (6225), 967-970.

(25) Stranks, S. D.; Eperon, G. E.; Grancini, G.; Menelaou, C.; Alcocer, M. J. P.; Leijtens, T.; Herz, L. M.; Petrozza, A.; Snaith, H. J. Electron-Hole Diffusion Lengths Exceeding 1 Micrometer in an Organometal Trihalide Perovskite Absorber. *Science* **2013**, *342* (6156), 341-344.

(26) Yuan, Y.; Yan, G.; Dreessen, C.; Rudolph, T.; Hülsbeck, M.; Klingebiel, B.; Ye, J.; Rau, U.; Kirchartz, T. Shallow defects and variable photoluminescence decay times up to 280 µs in triple-cation perovskites. *Nat. Mater.* **2024**, *23*, 391-397.

(27) Wu, B.; Wang, A.; Fu, J.; Zhang, Y.; Yang, C.; Gong, Y.; Jiang, C.; Long, M.; Zhou, G.; Yue, S. Uncovering the mechanisms of efficient upconversion in two-dimensional perovskites with anti-Stokes shift up to 220 meV. *Science Advances* **2023**, *9* (39), eadi9347.

(28) Granados del Águila, A.; Do, T.; Xing, J.; Jee, W. J.; Khurgin, J. B.; Xiong, Q. Efficient up-conversion photoluminescence in all-inorganic lead halide perovskite nanocrystals. *Nano Research* **2020**, *13* (7), 1962-1969.

(29) Lytle, K. M.; Brass, E. L.; Roman, B. J.; Sheldon, M. T. Thermal Activation of Anti-Stokes Photoluminescence in CsPbBr3 Perovskite Nanocrystals: The Role of Surface Polaron States. *ACS nano* **2024**, *18* (28), 18457-18464.

(30) Pokryshkin, N. S.; Mantsevich, V. N.; Timoshenko, V. Y. Anti-Stokes Photoluminescence in Halide Perovskite Nanocrystals: From Understanding the Mechanism towards Application in Fully Solid-State Optical Cooling. *Nanomaterials* **2023**, *13* (12), 1833.

(31) Qian, Q.; Wan, Z.; Takenaka, H.; Keum, J. K.; Smart, T. J.; Wang, L.; Wang, P.; Zhou, J.; Ren, H.; Xu, D. Photocarrier-induced persistent structural polarization in soft-lattice lead halide perovskites. *Nat. Nano.* **2023**, *18*, 357-364.

(32) Bisquert, J. Hysteresis, Impedance, and Transients Effects in Halide Perovskite Solar Cells and Memory Devices Analysis by Neuron‐Style Models. *Adv. Energy Mater.* **2024**, 2400442.

(33) Gu, C.; Lee, J.-S. Flexible hybrid organic–inorganic perovskite memory. *ACS nano* **2016**, *10* (5), 5413-5418.
